# Fall risk assessment scale for hemodialysis patients (ERQUE_HD): development and validation

**DOI:** 10.1590/2175-8239-JBN-2025-0283en

**Published:** 2026-06-05

**Authors:** Veronica Alacarini Farina, Simone Travi Canabarro, Gabriela de Souza, Lucas Teixeira Negrello, Ana Elizabeth Prado Lima Figueiredo

**Affiliations:** 1 Universidade Federal de Ciências da Saúde de Porto Alegre, Porto Alegre, RS, Brazil.; 2 Pontifícia Universidade Católica do Rio Grande do Sul, Porto Alegre, RS, Brazil.

**Keywords:** Accidental Falls, Patient Safety, Renal Dialysis, Risk Factors, Nursing

## Abstract

**Introduction:**

Hemodialysis patients present multiple comorbidities and complications during therapy, factors that increase the risk of falls. Adequate risk assessment is essential for implementing effective preventive measures.

**Objective:**

To develop and validate a scale for assessing fall risk in hemodialysis patients.

**Method:**

A methodological study with quantitative and qualitative approaches, conducted in five stages: integrative review, focus groups, instrument development, content validation, and scale validation.

**Results:**

The integrative review included 17 articles and supported the initial item development. Nine focus groups with patients and healthcare professionals enabled the identification of additional risk factors, resulting in the construction of the ERQUE_HD (Fall Risk Assessment Scale for Hemodialysis Patients), composed of 17 items distributed across four domains: health history, dialysis-related factors, functional capacity, and nutrition. Content validation reached a content validity index (CVI) of 0.95, considered highly satisfactory. The scale was then applied to 66 patients and analyzed using logistic regression with generalized estimating equations (GEE), including variables such as post-dialysis fatigue, interdialytic weight gain, and intradialytic hypotension. Patients classified as high-risk were 8.7 times more likely to experience falls compared to the moderate-risk group. The ERQUE-HD demonstrated good accuracy (AUC = 0.817). Post-dialysis fatigue and interdialytic weight gain >4% were identified as significant fall predictors.

**Conclusion:**

The ERQUE-HD proved to be effective in assessing and predicting fall risk among hemodialysis patients.

## Introduction

The demands associated with safety and quality of care have increased and spread worldwide over recent decades, generating substantial efforts to improve the patient safety culture in healthcare institutions^1^. According to the Global Action Plan, a new definition of patient safety has been established: a structured set of activities that promotes cultures, processes, procedures, behaviors, technologies, and environments in healthcare delivery that consistently and sustainably reduce risks, minimize the occurrence of preventable harm, make errors less likely, and reduce the impact of harm when it occurs^2^.

One of the patient safety protocols focuses on the prevention of falls, which may occur both in hospitalized patients and in those receiving outpatient care. Individuals with chronic kidney disease frequently require dialysis, with hemodialysis (HD) being one of the most commonly used modalities. Annual studies conducted in Brazilian dialysis centers indicate that, in July 2023, the total number of patients undergoing dialysis was 157,357, and 51,153 patients initiated the therapy in the same year^3^.

Hemodialysis (HD) can be performed in a hospital setting or in outpatient clinics, where the patient undergoes the therapy and is discharged after the procedure. It is considered a highly complex therapy due to the need for specialized equipment, the performance of invasive procedures to obtain vascular access, and the use of medications, such as anticoagulants, during the sessions^4^.

Given this context, it is essential to mitigate the risks of potential adverse events (AEs) that may occur in patients undergoing hemodialysis, such as abrupt reductions in blood pressure, vascular access infections, medication errors, and falls. Patients requiring this therapy are often in unfavorable clinical conditions and present multiple comorbidities, and any type of failure may result in serious consequences for their health^5^.

Although it is recognized that it is not possible to eliminate the risk of falls, an accurate assessment that considers individual characteristics and different patient profiles can significantly reduce this risk and enable the implementation of individualized measures for each patient^6,7^. In this context, the use of a validated scale is of great importance. However, the existing tools used to measure this risk were mostly developed in the 1990s and primarily focus on hospitalized clinical, geriatric, and surgical patients^8^.

Nevertheless, there are still no validated scales capable of adequately supporting the stratification of fall risk in adult patients undergoing hemodialysis, highlighting an important gap in care for this population.

The present study aimed to develop and validate a scale to assess the risk of falls in patients undergoing hemodialysis.

## Methods

**Figure 1:**
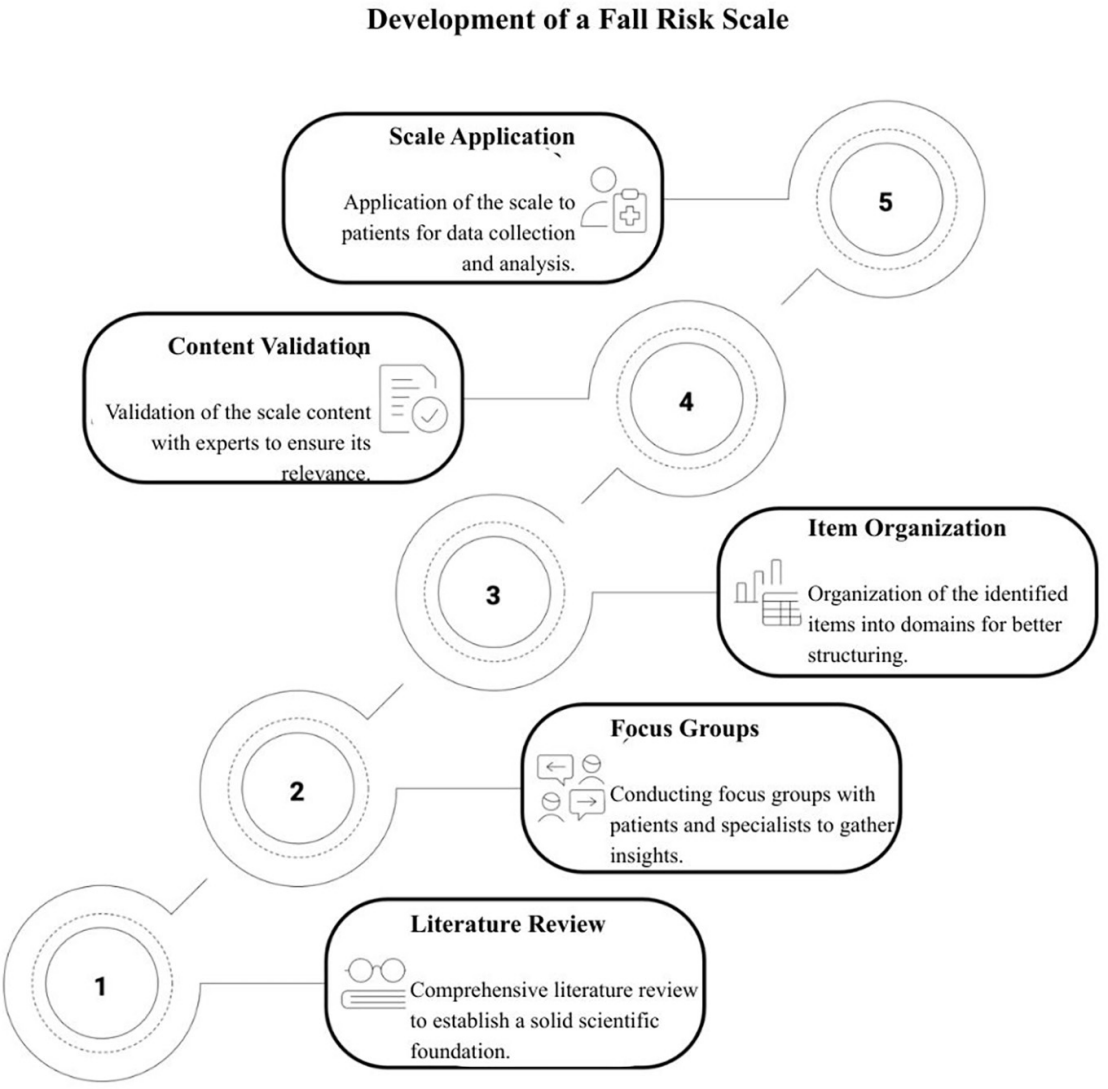
Stages of the ERQUE_HD scale development.

This is a methodological study with both quantitative and qualitative approaches, aimed at the development and validation of the scale entitled *Fall Risk Assessment Scale for Patients Undergoing Hemodialysis* (ERQUE_HD). Methodological studies support knowledge construction by seeking the most appropriate ways to measure a given phenomenon, whether through the development, validation, or evaluation of research tools and methods. In general, they are non-experimental in nature and focus on the creation of new instruments^9,10^. The project was approved by the Research Ethics Committee under CAAE number 65446322.5.0000.5345, in accordance with the requirements of *Plataforma Brasil*. The study was conducted following the stages described in Figure 1.

**Figure 2:**
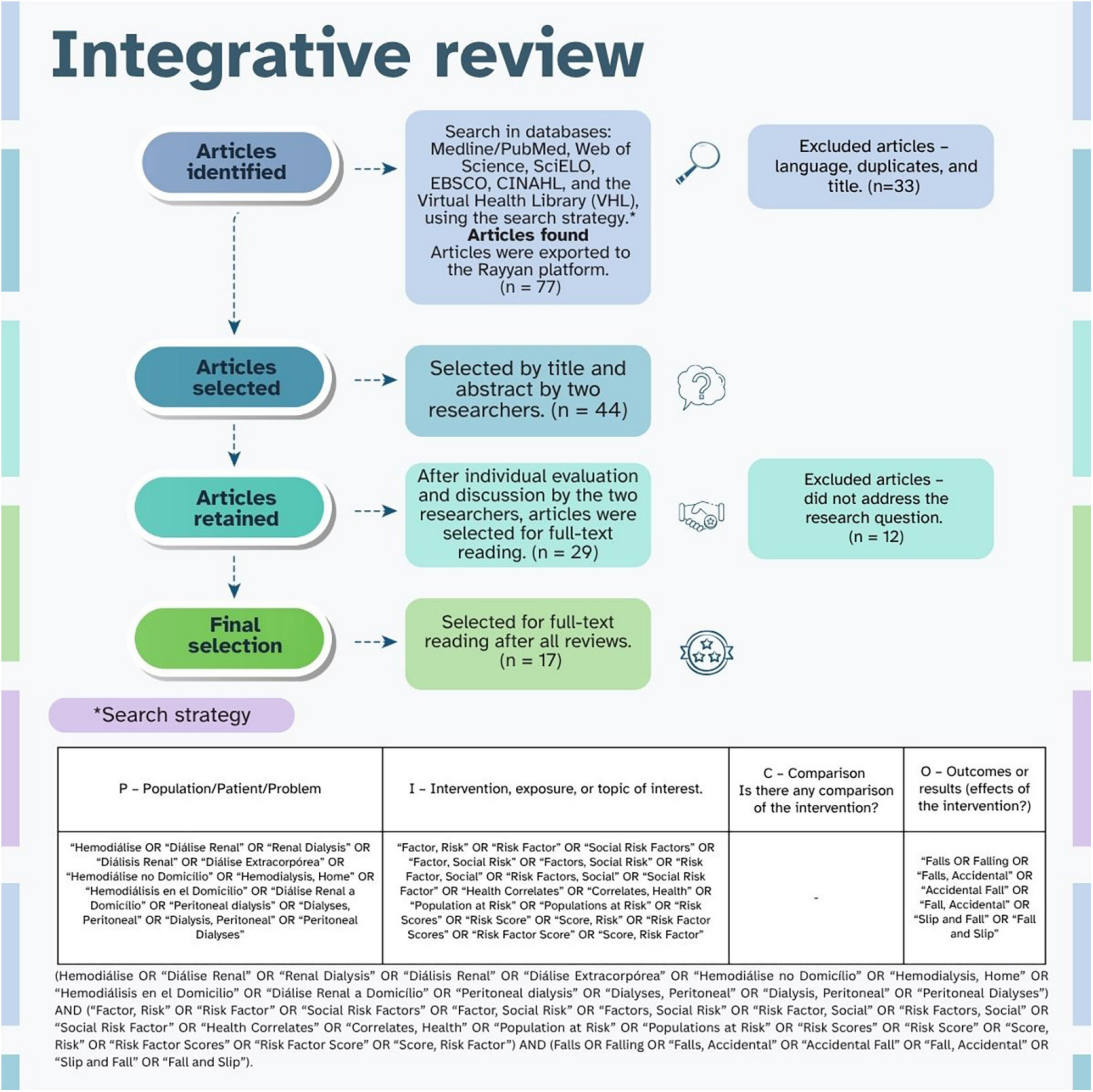
Integrative review process.

Each stage was developed as described below:

1. **Literature review:** The following steps were undertaken: 1) formulation of the guiding research question; 2) search and selection of primary studies in the literature; 3) data extraction from the studies; 4) critical appraisal of the primary studies included in the review; 5) synthesis of the review results; and 6) presentation of the method^11^. The guiding question was developed using the PICO strategy: “What are the main risk factors for falls in patients undergoing hemodialysis?”^12^. Figure 2 provides further details of the review process.
2. **Focus groups:** Nine focus groups were conducted, involving patients, nursing technicians from the two participating centers, and specialists from different healthcare institutions. Inclusion criteria for specialists were a minimum of a master’s degree in fields related to nephrology or patient safety and more than five years of professional experience in their area of practice, including care of hemodialysis patients, risk management, quality, and/or patient safety. The specialist focus groups included physicians, nurses, nutritionists, physical educators, and physiotherapists. Participants in the patient focus group were selected by convenience sampling, covering different age groups, all aged 18 years or older. Of the 16 participants, 11 were male and five were female, all undergoing hemodialysis therapy for at least two years and distributed across different treatment shifts, which allowed the inclusion of diverse profiles and lifestyles. For all groups, exclusion criteria were refusal to participate in the study or the presence of impairments that prevented understanding of the questions or participation in the discussions. Focus groups were conducted between April 2023 and May 2024. All participants who agreed to participate signed the Informed Consent Form (ICF) in person. Meetings were held face-to-face, with an average duration of 60 minutes, and all sessions were audio-recorded to ensure data reliability. Subsequently, all recordings were transcribed and reviewed by the principal investigator. In-person interaction facilitated the exchange of experiences and the generation of more in-depth insights.
3. **Item organization:** The items identified in the literature review were organized in a table and validated through an analysis of the focus group transcripts, based on the experiences reported. The items were then grouped into four thematic domains: health history, dialysis-related factors, functional capacity, and nutrition. The first version of the scale, consisting of 19 items, was developed. Subsequently, a new focus group with specialists evaluated the applicability of each item, resulting in adjustments and the finalization of the second version of the scale.
4. **Content validation:** Content validation was carried out by a committee of 15 patient safety specialists from various healthcare institutions, including physicians, pharmacists, and nurses with master’s and/or doctoral degrees. Participants were selected and invited using convenience sampling and completed the validation process virtually. All received an email invitation with the ICF attached for signature. For validation of the scale items, a Likert scale was used to score each item individually, along with a field for suggestions for improvement or additional comments^13,14^.
5. **Scale testing:** During the validation phase, the scale was applied to patients from different age groups and treatment shifts at one participating center. The scale was administered across three shifts (morning, afternoon, and evening), once per week to each participant, with consecutive applications over a four-week period. After application, the results were organized into a table and statistically analyzed. It is important to note that the item referring to falls in the past year was adapted to falls in the past week, as some patients had not been undergoing treatment for one year. This adaptation allowed better applicability of the item, as the scale was employed weekly, enabling identification of falls occurring between applications and the detection of changes in scores. Statistical analysis was performed using the Statistical Package for the Social Sciences (SPSS), version 25.0 (SPSS Inc., Chicago, IL, USA, 2018) for Windows. Results were presented using descriptive statistics, including absolute and relative distributions (n, %), as well as measures of central tendency (mean and median) and variability (standard deviation and interquartile range). The symmetry of continuous distributions was assessed using the Kolmogorov–Smirnov test. Comparisons of categorical variables across weeks were performed using the Friedman test. For comparisons between categorical variables and risk classifications and fall outcomes, Fisher’s exact test (Monte Carlo simulations) was applied. To assess the prediction of fall occurrence over the follow-up period as a function of risk classification and other relevant covariates, Poisson regression with robust variance was employed using Generalized Estimating Equations (GEE). Predictor analysis, expressed as odds ratios (OR), was initially conducted using univariate analysis, and variables with a p-value < 0.05 were included in the multivariate model. The Receiver Operating Characteristic (ROC) curve was constructed using the ERQUE_HD scale scores to discriminate between participants with and without falls, according to the classification estimated by the predictive model. The area under the curve (AUC) was reported with a 95% confidence interval. According to Hosmer and Lemeshow^15^, an AUC between 0.70 and 0.80 is considered “acceptable,” between 0.80 and 0.90 “excellent,” and above 0.90 “exceptional.” The Youden index (sensitivity + specificity − 1) was also calculated to determine the ERQUE_HD scale score with the best discriminative performance.

## Results

The results are described according to the stages of the study, following the same order presented in the Methods section.

**Figure 3:**
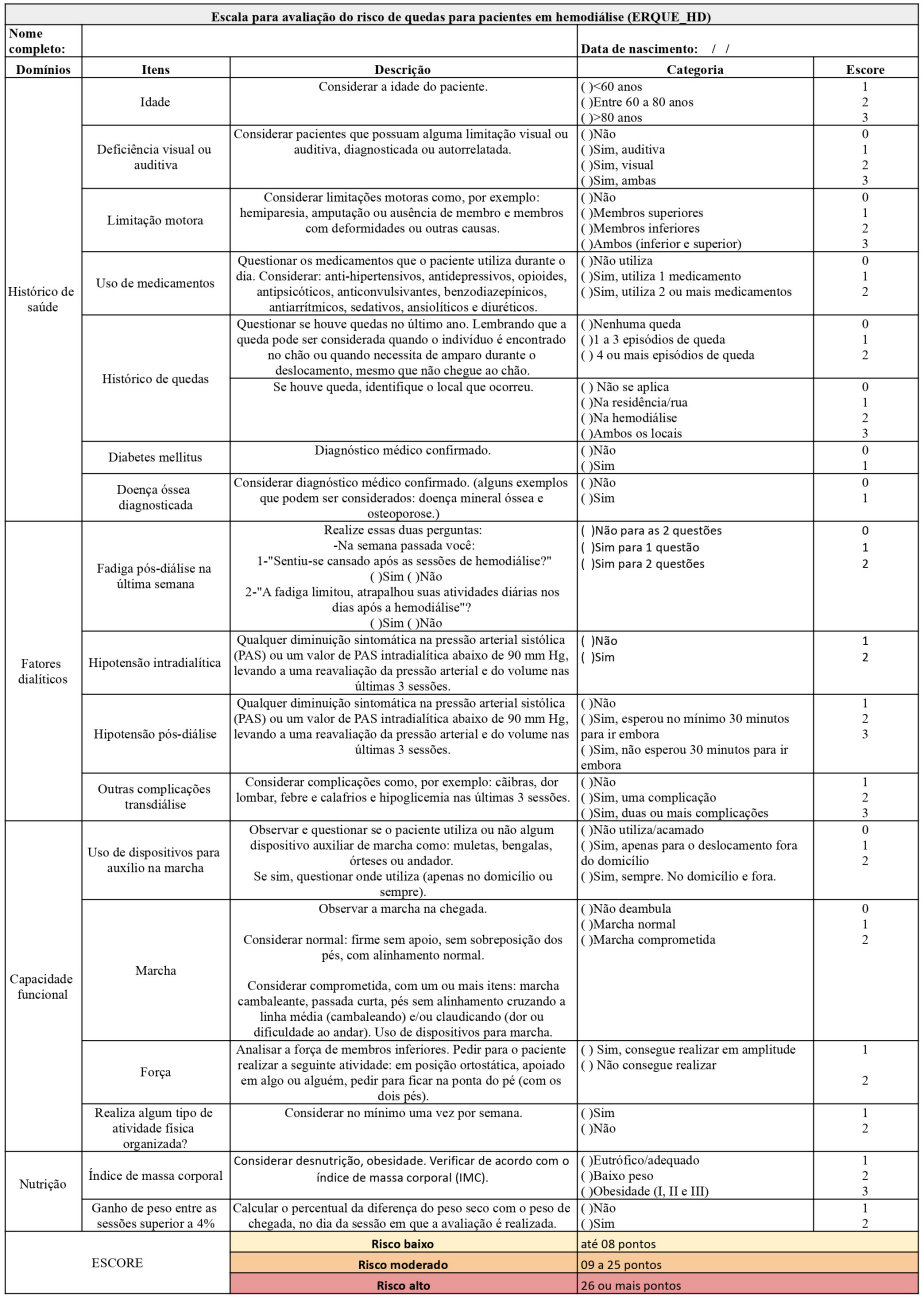
ERQUE_HD scale.

1. **Integrative review:** The identified articles were imported into the Rayyan platform for screening. Initially, 94 studies were retrieved from the Medline/PubMed, Web of Science, SciELO, EBSCO, CINAHL, and BVS databases. After the removal of 27 duplicates, 77 articles remained, of which 33 were excluded after title and abstract screening. Of the 44 selected studies, 29 were assessed in full, resulting in the final inclusion of 17 articles that met the review criteria. These articles were read in full and organized in a spreadsheet containing the main risk factors, as described in Table 1.
2. **Focus groups:** All focus group transcripts were carefully reviewed. The risk factors mentioned by participants were identified and categorized in spreadsheet format, facilitating visualization and comparative analysis. The relevant factors identified were: depression, anxiety, and age; one group cited female sex, whereas six groups cited male sex as a risk factor; visual impairment, hearing impairment, fatigue, hypoglycemia, need for walking assistance, postural hypotension, cramps during dialysis sessions, interdialytic weight gain, lack of physical exercise, bone diseases, use of medications (antihypertensive agents), muscle weakness, impaired postural balance, functional capacity, diabetes, hypertension, and history of falls.
3. **Instrument development:** By compiling the items from the integrative review and the factors addressed in all focus groups, the most relevant elements were grouped into specific domains and adjusted based on the discussions. Overall, 17 items were included, divided into four domains: health history, dialysis-related factors, functional capacity, and nutrition.
4. **Content validation:** For this stage, the content validity index (CVI) was calculated. The score was obtained by summing the ratings assigned to each item by the experts, considering those rated as “3” or “4.”^16^ Items rated as “1” or “2” required revision or exclusion, as they were classified as “1: not relevant” or “2: needs revision to assess relevance.” The scale was finalized, with its final version presented in Figure 3, comprising a total of 17 items. Considering the CVI values of the retained items, the mean CVI was 0.95, which reinforces that the content of the scale was validated according to the recommended values. As demonstrated in Table 2, of the initial 19 items, two were removed from the scale because they had values below the recommended threshold, namely, sex and appetite, which were not considered relevant.
5. **Scale validation:** The scale was applied in one of the participating centers over a four-week period, covering the three operating shifts. The results refer to an initial sample of 66 patients followed over this period. In week one (S1) and week two (S2), the total number of participants was maintained, although three patients discontinued follow-up due to death, relocation, or hospitalization and were replaced by three others. In week three (S3), a reduction of 9.1% (n = 6) in the sample was observed, resulting in 60 patients remaining, and in week four (S4), there was a further reduction of 10.6% (n = 7), resulting in a total of 59 participants at the end of follow-up.

**Table 1:** Summary of the selected articles with the main risk factors for falls

Title	Authors	Country/Year	Methodology	Objectives	Conclusions/Main factors leading to falls
Frailty and falls among adult patients undergoing chronic hemodialysis: a prospective cohort study^26^	Mara A McAdams-DeMarco; Sunitha Suresh; Andrew Law; Megan L Salter; Luis F Gimenez; Bernard G Jaar; Jeremy D Walston; Dorry L Segev.	United States, 2013	Longitudinal study	To identify whether frailty is associated with falls, regardless of other fall risk factors, in hemodialysis patients of all ages.	Frailty; advanced age; comorbidities; medication use.
Association of Self-Reported Frailty with Falls and Fractures among Patients New to Dialysis^27^	Cynthia Delgado; Stephanie Shieh; Barbara Grimes; Glenn M. Chertow; Lorien S. Dalrymple; George A. Kaysen; John Kornak; and Kirsten L. Johansen.	United States, 2015	Prospective cohort study	To examine the rates of falls or fractures requiring medical consultation or hospitalization in a diverse cohort of patients enrolled in the Comprehensive Dialysis Study, a special study of the U.S. Renal Data System.	Frailty; history of falls.
The effect of hemodialysis on balance measurements and risk of fall^28^	Erken E; Ozelsancak R; Sahin S; Yılmaz EE; Torun D; Leblebici B; Kuyucu YE; Sezer S.	Turkey, 2016	Case-control study	To investigate the effect of hemodialysis treatment on balance and explore the possible association between hemodialysis and an increased incidence of fall-related injuries in patients with ESRD.	Fatigue; age; low hemoglobin; hypotension; polypharmacy; inadequate dialysis; electrolyte disturbances.
Association of Reduced eGFR and Albuminuria with Serious Fall Injuries among Older Adults^29^	C Barrett Bowling; Samantha G Bromfield; Lisandro D Colantonio; Orlando M Gutiérrez; Daichi Shimbo; Kristi Reynolds; Nicole C Wright; Jeffrey R Curtis; Suzanne E Judd; Harold Franch; David G Warnock; William McClellan; Paul Muntner.	United States, 2016	Prospective cohort study	To determine the association of reduced eGFR and elevated albumin-to-creatinine ratio (ACR) with the risk of serious fall-related injuries, defined as fractures, brain injury, or joint dislocation.	Low eGFR levels.
Development and validation of a Fall Risk Assessment Index for dialysis patients^30^	Kono K; Nishida Y; Yabe H; Moriyama Y; Mori T; Shiraki R; Sato T.	Japan, 2017	Epidemiological cohort study	To develop a comprehensive symptom assessment tool to predict falls with high sensitivity in dialysis patients.	Advanced age; inflammatory status; malnutrition; hypotension; balance impairment; history of falls.
Serious Fall Injuries Before and After Initiation of Hemodialysis Among Older ESRD Patients in the United States: A Retrospective Cohort Study ^31^	Plantinga LC; Patzer RE; Franch HA; Bowling CB.	United States, 2017	Retrospective cohort study	To determine the rate of serious fall-related injuries among older hemodialysis patients and whether this rate differs before and after initiation of dialysis therapy.	Older age; post-hemodialysis period compared to pre-hemodialysis.
The prevalence and impact of falls in elderly dialysis patients: Frail Elderly Patient Outcomes on Dialysis (FEPOD) study^32^	Ismay N. van Loon; Hanneke Joosten; Osasuyi Iyasere; Lina Johansson; Marije E. Hamaker; Edwina A. Brown.	United Kingdom, 2019	Observational longitudinal study	To assess the prevalence of falls and fractures in frail older adults on dialysis in the FEPOD study, their association with mortality and hospitalization, and their impact on functional performance and quality of life (QoL).	Frailty; diabetes; quality of life; advanced age.
Risk of falls in people with chronic kidney disease and related factors^23^	Thaís Carrera de Carvalho; Ariane Polidoro Dini.	Brazil, 2020	Quantitative, descriptive, and correlational study	To identify the risk and prevalence of falls in the past year among chronic kidney disease patients on hemodialysis; associate fall risk with fear of falling and sociodemographic variables.	Diabetes; use of walking aids; use of orthoses.
Risk of Serious Falls Between Hemodialysis and Peritoneal Dialysis Patients: A Nationwide Population-based Cohort Study^33^	Wang HH; Wu JL; Lee YC; Ho LC; Chang MY; Liou HH; Hung SY.	China, 2020	Cohort study	To estimate the risk of serious falls among patients on HD and PD to determine which modality is more strongly associated with severe fall events.	Advanced age; cardiovascular problems; history of falls prior to dialysis; female sex; stroke; age-related physical, sensory, and cognitive changes; use of diuretics, blockers, and mydriatics; frailty; post-dialysis fatigue; malnutrition; gait speed.
The relative importance of frailty, physical and cardiovascular function as exercise-modifiable predictors of falls in haemodialysis patients: a prospective cohort study^34^	Zanotto T; Mercer TH; van der Linden ML; Rush R; Traynor JP; Petrie CJ; Doyle A; Chalmers K; Allan N; Shilliday I; Koufaki P.	United Kingdom, 2020	Prospective observational study	To explore the relative importance of frailty and cardiovascular function as potential exercise-modifiable predictors of falls in patients with CKD stage 5 on hemodialysis.	Frailty; handgrip strength; daily steps; gait speed; Timed Up and Go (TUG); cardiovascular function; hypotension; low baroreflex effectiveness index (BEI); antihypertensive medications.
Markers of protein-energy wasting and physical performance in haemodialysis patients: a cross-sectional study^35^	Vanden Wyngaert K; Celie B; Calders P; Eloot S; Holvoet E; Van Biesen W; Van Craenenbroeck AH.	Belgium, 2020	Cross-sectional study	To assess the cross-sectional relationship between nutritional status, muscle strength, exercise capacity, and fall risk.	Nutritional status; frailty; lower limb muscle strength.
Factors associated with falls in hemodialysis patients: a case-control study^36^	Ignacio Perez-Gurbindo; Ana Maria Álvarez-Méndez; Rafael Pérez García; Patrícia Arribas-Cobo; Maria Teresa Angulo-Carrére.	Spain, 2021	Case-control study	To identify possible associations between increased likelihood of falls in hemodialysis patients and laboratory parameters, comorbidities, pharmacological treatment, hemodynamic changes, dialysis outcomes, and stabilometric alterations.	Intradialytic weight gain (>1.9 kg); patients with limited chronotropic response due to intrinsic or extrinsic causes are at higher risk of falls when undergoing HD; antihypertensives; higher β2-microglobulin levels.
Dysfunction in dynamic, but not static balance is associated with risk of accidental falls in hemodialysis patients: a prospective cohort study^37^	NobuyukiShirai; Suguru Yamamoto; Yutaka Osawa; Atsuhiro Tsubaki; Shinichiro Morishita; Ichiei Narita.	Japan, 2022	Prospective observational study	To evaluate the details of accidental falls and their association with balance function in hemodialysis patients.	Advanced age; association with the Timed Up and Go (TUG) test; active older men with low physical function, more likely; dynamic balance impairment.
Relationship between Nutrition-Related Problems and Falls in Hemodialysis Patients: A Narrative Review^38^	Shirai N; Inoue T; Ogawa M; Okamura M; Morishita S; Suguru Y; Tsubaki A.	Japan, 2022	Narrative review	To summarize the impact of frailty, sarcopenia, malnutrition, protein-energy wasting (PEW), and cachexia on falls in hemodialysis patients.	Frailty; malnutrition; muscle atrophy; postural balance impairment; hypotension; fluid removal; age; male sex; history of falls; antidepressant use; polypharmacy; low quality of life; lower limb muscle strength and physical function; diabetes; elevated parathyroid hormone; elevated C-reactive protein (CRP).
Novel risk-factor analysis and risk-evaluation model of falls in patients receiving maintenance hemodialysis^39^	Liu X; Chen S; Liu C; Dang X; Wei M; Xin X; Gao J.	China, 2022	Cross-sectional study	To investigate the prevalence of falls in patients with CKD on hemodialysis, analyze associated risk factors, and develop a risk assessment model.	Age; hearing impairment; use of walking aids; anxiety; depression; weakness.
Incidence and risk factors of falls in patients undergoing hemodialysis: a multicenter survey in northern China^40^	Liang J; Wang Y; Zhang W; Ding H; Gao Y; Wang R; Sun X; Peng Y; Gan L; Zuo L.	China, 2023	Prospective longitudinal study	To provide a clear outline of the incidence of falls in northern China and identify associated risk factors for falls in hemodialysis patients across all age groups.	Advanced age (related to frailty); anemia (related to fatigue, weakness, and low energy); gait and functional abnormalities.
Walking aids and complicated orthopedic diseases are risk factors for falls in hemodialysis patients: an observational study^41^	Ishii T; Matsumoto W; Hoshino Y; Kagawa Y; Iwasaki E; Takada H; Honma T; Oyama K.	Japan, 2023	Observational study	To statistically explore factors associated with accidental falls in dialysis facilities for future fall prevention.	Use of walking aids; history of falls; visual impairment; urinary incontinence; functional limitation; older adults; use of mobility aids.

**Table 2:** Scale items according to the content validity index (CVI)

Item	CVI	Scale classification
1. Sex	0.53	Item removed
2. Age	1.00	Item retained
3. Visual and hearing impairment	1.00	Item retained
4. Motor limitation	1.00	Item retained
5. Use of medications	1.00	Item retained
6. History of falls	1.00	Item retained
7. Diabetes mellitus	0.86	Item retained
8. Diagnosed bone disease	0.86	Item retained
9. Post-dialysis fatigue in the past week	0.93	Item retained
10. Intradialytic hypotension	1.00	Item retained
11. Post-dialysis hypotension	1.00	Item retained
12. Other intradialytic complications	0.93	Item retained
13. Use of gait-assistive devices	1.00	Item retained
14. Gait	1.00	Item retained
15. Strength	1.00	Item retained
16. Do you engage in any type of organized physical activity?	0.86	Item retained
17. Body mass index	0.93	Item retained
18. Appetite	0.60	Item removed
19. Interdialytic weight gain greater than 4%	0.86	Item retained
Sum of retained items (CVI)	16.23/17 = 0.95	

Table 3 presents the characterization of the sample in each week, considering variations in the number and composition of the patients followed. According to the results, no statistically significant differences were detected when comparing the investigated variables across the weeks. Thus, although variations in the patient sample and in the reported responses occurred, these were not considered relevant, indicating that the weeks showed similar characteristics regarding the investigated variables.

**Table 3:** Absolute and relative distribution of the general sample characteristics by follow-up week

Sample characteristics	Weeks^a^								p^b^
	Week 1 – S1 (n = 66)		Week 2 – S2 (n = 66)		Week 3 – S3 (n = 60)		Week 4 – S4 (n = 59)		
	n	%	n	%	n	%	n	%	
Age									0.516
60–80 years (2)	39	59.1	34	51.5	36	60.0	31	52.5	
>80 years (3)	7	10.6	9	13.6	6	10.0	9	15.3	
<60 years (1)	20	30.3	23	34.8	18	30.0	19	32.2	
Visual or hearing impairment									0.122
No (0)	27	40.9	33	50.0	37	61.7	21	35.6	
Yes, both (3)	7	10.6	7	10.6	5	8.3	3	5.1	
Yes, hearing (1)	4	6.1	2	3.0	1	1.7	1	1.7	
Yes, visual (2)	28	42.4	24	36.4	17	28.3	34	57.6	
Motor limitation									0.711
Both			2	3.0					
Lower limbs (2)	18	27.3	17	25.8	12	20.0	12	20.3	
Upper limbs (1)	1	1.5							
No (0)	47	71.2	47	71.2	48	80.0	47	79.7	
Medication use									0.822
Does not use (0)	3	4.5			2	3.3			
Yes, uses 1 medication (1)	1	1.5	1	1.5	1	1.7			
Yes, uses 2 medications (2)	62	93.9	65	98.5	57	95.0	59	100.0	
Diabetes Mellitus									0.942
No (0)	39	59.1	42	63.6	36	60.0	36	61.0	
Yes (1)	27	40.9	24	36.4	24	40.0	23	39.0	
Bone disease									0.879
No (0)	60	90.9	61	92.4	55	91.7	56	94.9	
Yes (1)	6	9.1	5	7.6	5	8.3	3	5.1	
Post-dialysis fatigue: “Did you feel tired after hemodialysis sessions?” “Did fatigue limit or interfere with your daily activities on the days following hemodialysis?”									0.758
No to both questions (0)	17	25.8	18	27.3	14	23.3	17	28.8	
Yes to one question (1)	17	25.8	19	28.8	21	35.0	17	28.8	
Yes to both questions (2)	32	48.5	29	43.9	25	41.7	25	42.4	
Intradialytic hypotension									0.073
No (0)	27	40.9	32	48.5	35	58.3	37	62.7	
Yes (1)	39	59.1	34	51.5	25	41.7	22	37.3	
Post-dialysis hypotension									0.376
No (1)	36	54.5	30	45.5	36	60.0	39	66.1	
Yes, waited at least 30 minutes before leaving (2)	28	42.4	29	43.9	22	36.7	18	30.5	
Yes, did not wait 30 minutes before leaving (3)	2	3.0	7	10.6	2	3.3	2	3.4	
Other complications (cramps, low back pain, fever, chills, hypoglycemia)									0.206
No (1)	15	22.7	21	31.8	18	30.0	19	32.2	
Yes, ≥2 complications (3)	18	27.3	12	18.2	4	6.7	4	6.8	
Yes, one complication (2)	33	50.0	33	50.0	38	63.3	36	61.0	
Use of walking aid									
Does not use/bedridden (0)	50	75.8	49	74.2	48	80.0	48	81.4	0.447
Yes, only outside the home (1)	3	4.5	3	4.5	1	1.7	1	1.7	
Yes, always (home and outside) (2)	13	19.7	14	21.2	11	18.3	10	16.9	
Gait									0.156
Impaired gait (2)	14	21.2	18	27.3	11	18.3	17	28.8	
Normal gait (1)	49	74.2	46	69.7	45	75.0	40	67.8	
Non-ambulatory (0)	3	4.5	2	3.0	4	6.7	2	3.4	
Strength – standing on tiptoes									0.167
Unable to perform (2)	21	31.8	26	39.4	15	25.0	19	32.2	
Yes, able to perform with full range (1)	45	68.2	40	60.6	45	75.0	40	67.8	
Body mass index (BMI)									0.142
Normal (1)	25	37.9	21	31.8	25	41.7	18	30.5	
Underweight (2)	8	12.1	10	15.2	6	10.0	8	13.6	
Obesity class I, II, or III (3)	33	50.0	35	53.0	29	48.3	33	55.9	
Do you engage in any physical activity?									0.305
No (2)	44	66.7	47	71.2	38	63.3	41	69.5	
Yes (1)	22	33.3	19	28.8	22	36.7	18	30.5	
Interdialytic weight gain >4%									0.183
No (1)	32	48.5	26	39.4	27	45.0	31	52.5	
Yes (2)	34	51.5	40	60.6	33	55.0	28	47.5	
Age (years)									
Mean ± standard deviation	63.6 ± 15.3								
Duration of dialysis (years)									
Mean ± standard deviation (range)	4.5 ± 5.3								
Median (1st–3rd quartile)	2.3 (0.8–7.0)								
Type of catheter									
Central double-lumen catheter	31		47.0	32	48.5	30	50.0	28	47.5
Arteriovenous fistula	35	53.0	34	51.5	30	50.0	31	52.5	

Notes – ^a^: Percentages obtained based on the total for each week.

^b^: Friedman test.

Information on falls and risk classification according to the scale was also analyzed. In S1, 40.9% of patients reported one to three falls in the past year, and 30.3% reported four or more episodes, with no statistically significant differences between the weeks. Most falls occurred at home or on streets, ranging from 51.5% to 55.9%. Regarding risk classification using the ERQUE_HD, moderate risk predominated in all weeks, with proportions ranging from 80.3% to 83.3%. Concerning falls in the past week, there was no statistically significant variation throughout the follow-up (p = 0.366); most patients did not experience falls during this period, ranging from 71.7% in S3 to 79.7% in S4. Among those who reported recent falls, episodes of one to three events were the most common, accounting for up to 19.7% of cases. In terms of fall risk classification in the past week, most respondents were classified as moderate risk.

An attempt was made to identify how the general characteristics of the sample were related to the occurrence of falls. Considering the original classification of fall occurrence, both in the past year and in the past week, statistically significant results were identified.

Analyses of fall-related outcomes showed significant associations between events in the past year and several clinical variables. In S3, visual and hearing impairment were associated with more episodes (≥4). Post-dialysis fatigue showed a significant association in S1: patients without fall events reported less fatigue, whereas those with more episodes reported greater limitations in activities after dialysis. Intradialytic hypotension was strongly associated with falls in weeks S2 and S4, especially among those who experienced four or more episodes. Post-dialysis hypotension was significant in S2 and occurred more frequently among affected patients. In S3, impaired gait was associated with a higher number of events, while preserved gait was observed among those with fewer episodes. Finally, interdialytic weight gain (>4%) was associated with the occurrence of falls in weeks S3 and S4, being more common among patients who reported such episodes, whereas those without falls maintained stable weight between sessions.

The absolute and relative distributions of the characteristics that showed statistically significant differences when compared with fall episodes in the past week were analyzed. The results revealed statistically significant associations with several clinical factors. Intradialytic hypotension was strongly associated with fall occurrence in weeks S3 and S4: patients who did not experience falls were associated with the absence of hypotension—67.4% (n = 29) and 72.3% (n = 34), respectively—while those with fall episodes, especially the ones with four or more, showed a strong association with this condition. In S3 and S4, patients with four or more episodes showed a 100.0% (n = 6) association with the presence of hypotension. Post-dialysis hypotension was also related to the occurrence of falls in the past week, in S3, being more common among patients who reported this event. The use of gait-assistive devices was significantly associated with fall occurrence, particularly in weeks S2 and S3, especially among those who reported constant use of these devices. In addition, impaired gait was also associated with falls in S3, while normal gait was predominant in patients who did not experience falls.

To assess the relationship between ERQUE_HD risk classification and the occurrence of falls, comparisons were performed for each week, considering both fall episodes in the past year and in the past week. Initially, the events were analyzed in three categories and subsequently grouped into a dichotomous variable (yes/no). The results indicated a statistically significant association between risk classification and fall occurrence in the past year. Patients classified as moderate risk were mostly associated with the absence of events, while those classified as high risk showed a significant association with four or more episodes. This association remained significant even after dichotomization of the variable, reinforcing that high-risk classification is related to fall occurrence in the past year, as shown in Table 4.

**Table 4:** Absolute and relative distribution of falls in the past year by risk classification

Falls in the past year	ERQUE_HD risk classification (scale scores – past year)^a^				p^b^
	Moderate risk		High risk		
	n	%	n	%	
Week 1					0.005
No falls	19	35.8			
1–3 episodes	22	41.5	5	38.5	
≥4 episodes	12	22.6	8	61.5	
Week 2					0.002
No falls	19	34.5			
1–3 episodes	25	45.5	3	27.3	
≥4 episodes	11	20.0	8	72.7	
Week 3					0.005
No falls	15	30.0			
1–3 episodes	21	42.0	2	20.0	
≥4 episodes	14	28.0	8	80.0	
Week 4					0.30
No falls	14	28.6			
1–3 episodes	22	44.9	3	30.0	
≥4 episodes	13	26.5	7	70.0	
FALL YES/NO in the past year					
Week 1					0.013
No	19	35.8			
Yes	34	64.2	13	100.0	
Week 2					0.026
No	19	34.5			
Yes	36	65.5	11	100.0	
Week 3					0.054
No	15	30.0			
Yes	35	70.0	10	100.0	
Week 4					0.098
No	14	28.6			
Yes	35	71.4	10	100.0	

Notes – ^a^Percentages calculated based on the total for each classification of fall occurrence over the past year.

^b^Fisher’s exact test (Monte Carlo simulation).

Another analysis compared fall episodes in the past week with risk classification, and significant associations were observed. Patients classified as high risk showed a significant association with four or more fall events in the past year, with results evident in weeks S2 and S3. When the comparison was performed between risk classification and falls using a dichotomous response, a significant difference was observed in all three weeks evaluated.

To evaluate the predictive capacity of the scale regarding the occurrence of falls in the past year, GEE modeling with logistic regression adjusted for the four-week follow-up period was used. The outcome considered was fall occurrence (yes/no), and the model also investigated the impact of covariates associated with this outcome or risk classification. Among the predictors with potential significance were visual/hearing impairment, post-dialysis fatigue (p <0.001), intradialytic hypotension, post-dialysis hypotension, impaired gait, and weight gain greater than 4%. Initially, individual models were tested for each predictor to analyze their isolated contribution to explaining falls in the past year.

Bivariate analysis of univariate logistic regression models, using the GEE method to predict/explain the occurrence of falls in the past year (OR and 95%CI), demonstrated that ERQUE_HD risk classification has significant predictive capacity for falls in the past year (p < 0.0001). Patients classified as high risk had 6.764-fold higher odds of falling compared with those at moderate risk. Among the covariates with predictive potential, visual/hearing impairment stood out (p = 0.006), increasing fall risk by 1.856 times, and post-dialysis fatigue (p = 0.003), which increased the risk by 5.588 times among patients who answered positively to both fatigue-related questions. Post-dialysis hypotension was also significant, with patients who waited at least 30 minutes before leaving presenting 2.696-fold higher odds of falling. Although hypotension without waiting suggested an increased risk, the result was not statistically significant, likely due to the small number of cases. Interdialytic weight gain greater than 4% was another relevant factor (p < 0.001), increasing the odds of falls by 2.292-fold. Finally, intradialytic hypotension and gait variables did not remain significant, possibly due to a loss of effect over the follow-up period.

Based on the results obtained from the univariate models, variables that showed significant results in predicting/explaining the occurrence of falls were selected for inclusion in the multivariate model. Thus, the initial multivariate model included ERQUE_HD risk classification, visual/hearing impairment, bone disease, fatigue, post-dialysis hypotension, and weight gain. According to the results, the optimal model identified as a predictor of fall occurrence in the past year included the following predictors: risk classification (high risk—OR; 95% CI), post-dialysis fatigue (yes to both questions—OR; 95% CI), and weight gain greater than 4% between sessions (yes—OR; 95% CI).

Notably, risk classification, as a predictor of the occurrence of falls, showed increased predictive power in the presence of fatigue and interdialytic weight gain exceeding 4%. In the simple model, the OR for high risk was estimated at 6.764, whereas in the multivariate model, the OR increased to 8.731, indicating an enhanced predictive performance of the risk classification variable, as shown in Table 5.

**Table 5:** Multivariate logistic regression model using GEE to predict/explain the occurrence of falls in the past year

Sample characteristics	OR for the occurrence of falls in the past year				
	Odds ratio^a^				Odds ratio^a^
	OR	95% CI^b^			
MODEL 2c					
Annual risk classification					
Moderate risk	1.0				
High risk	8.731	2.681	19.687		<0.00001
Post-dialysis fatigue: “Did you feel tired after hemodialysis sessions?” ”Did fatigue limit or interfere with your daily activities on the days following hemodialysis?”					
No to both questions (0)	1.0				
Yes to one question (1)	3.113	1.135	8.545		0.053
Yes to both questions (2)	5.715	1.841	17.743		0.003
Interdialytic weight gain >4%					
No (1)	1.0				
Yes (2)	2.415	1.161	5.026		<0.001

Notes – ^a^: Estimates obtained using Generalized Estimating Equations (GEE).

^b^:OR, Odds ratio; 95% CI, 95% confidence interval for the OR.

^c^:Logistic model performance – confusion matrix (overall accuracy: 78.9%; correct classification of fall occurrence: 95.7% [176/184]; correct classification of non-occurrence of falls: 32.8% [45/67]). Cox & Snell R^2^: 0.288. Nagelkerke R2: 0.419.

Regarding model performance, the robustness of the classification accuracy achieved by logistic regression was investigated in relation to the actual classification of falls observed in the past year. The area under the ROC curve was used; in addition to estimating the AUC to identify statistical significance of the model’s predictive capacity, sensitivity and specificity values were also reported.

According to the results, the AUC was estimated at 0.817 (95% CI: 0.745–0.889; p <0.001). In the analysis of sensitivity and specificity of the predictive model for falls in the past year, the estimates were 0.876 and 0.684, respectively. It is noteworthy that ROC curve estimates were based on the ERQUE_HD scale scores according to the predictive model classification, with the cutoff point for fall occurrence set at scores greater than 23 (cutoff point > 23).

The analysis involving the accuracy achieved by the univariate model, which used risk classification alone to predict the occurrence of falls, yielded a ROC curve estimate of 0.702 (95% CI: 0.614–0.811). These results indicate that the accuracy was also relevant for predicting fall episodes in the past year. Sensitivity and specificity estimates for the univariate predictive model were 0.814 and 0.568, respectively.

## Discussion

The development and validation of the ERQUE_HD represent a complex process, requiring scientific knowledge combined with clinical outcomes and a broad range of expertise from specialists and the population undergoing this therapy. The results of this study identified several fall risk factors specific to the target population. After multiple stages, the items were defined, culminating in the final version of the scale, with a CVI of 0.95, reinforcing that the content of the scale was validated according to the recommended values^9,13^.

The relevance of this instrument becomes even more evident in the context of global population aging. Within this scenario, age was included as a risk factor in the scale based on evidence identified in the focus groups and the integrative review. Reinforcing this context, the World Health Organization (WHO) reports that by 2050 more than 2 billion people worldwide will be over 60 years of age^17^. It is estimated that within a twelve-month period, one-third of the population aged 65 years or older will experience at least one fall, and half of these individuals will have recurrent episodes^18^.

In this context, according to the 2023 Brazilian Dialysis Census, 62.5% of patients undergoing hemodialysis were between 20 and 64 years of age, and the remaining 33.6% were 65 years of age or older^3^. When analyzing the results of the present study, it was observed that 42.03% of the participants undergoing hemodialysis were aged 65 years or older, a proportion higher than that reported by the census. This discrepancy may be partly attributed to the smaller sample size of the current study compared with that of the population evaluated in the census.

This study revealed that 76% of participants undergoing hemodialysis experienced falls in the past year, a percentage substantially higher than estimates reported in the literature, such as a study that reported an incidence of 23.3%^19^. This difference can largely be attributed to the methodology used in this study, particularly the active ascertainment of fall-related information through direct and detailed patient interviews.

In contrast, many studies in the literature rely on secondary data or spontaneous reporting, which may result in underreporting of events such as falls, especially when they do not lead to severe injuries or hospitalizations. Thus, the findings of this study reinforce the importance of active monitoring strategies and systematic investigation of falls among hemodialysis patients, as the high prevalence observed indicates a significant and potentially underrecognized health problem. One study reported that active surveillance resulted in a prevalence up to eight times higher than that obtained through spontaneous reporting, reiterating its importance as an epidemiological monitoring method for a more reliable situational diagnosis and as a fundamental tool in patient safety^20^.

Although studies indicate a strong association between the use of certain medications and increased risk of hypotension and falls—particularly antihypertensive agents and those acting on the central nervous system, such as antiparkinsonian drugs, psychotropics, psychoanaleptics, and antiepileptics^21^—there is no conclusive evidence of a significant correlation in clinical practice. Nevertheless, it is essential, whenever possible, to obtain a comprehensive review of the medications used by patients to enable more effective management. Although the ERQUE_HD does not assign specific scores to individual medications related to fall risk, 93.9% of participants used more than two medications daily. This reinforces the importance of including an item that broadly assesses medication use without assigning scores to specific drugs.

Regarding health history, previous studies indicate a strong association between diabetes and fall risk. One of the most common complications of diabetes is neuropathy—both sensory and motor—which may be characterized by symptoms such as loss of proprioception, atrophy, and weakness of the intrinsic foot muscles, leading to foot deformities and gait abnormalities, thereby significantly increasing fall risk^22^. However, after applying the ERQUE_HD scale, neither the presence of diabetes nor possible motor impairment—a factor related to disease progression—showed statistical significance when compared with other dialysis-related complications. Nevertheless, considering the analyzed studies and the focus group discussions, it was decided to assign a score to patients with a confirmed diagnosis of this condition.

A study evaluating patients with mineral bone disease secondary to chronic kidney disease found that these patients had a high risk of falls and greater impairment in physical aspects and quality of life^23^. Similarly, visual/hearing impairment and bone disease, when assessed in isolation, showed high statistical significance; however, when considered in conjunction with other risk factors, their relevance was significantly reduced. These factors were therefore included in the scale assessment.

Other complications may occur during therapy and be associated with additional fall risk factors, such as cramps, low back pain, fever, chills, hypoglycemia, and symptomatic intradialytic hypotension^24^. The occurrence of symptomatic hypotension likely depends on the amount and rate of intravascular fluid removal during the procedure. In response to ultrafiltration (UF), a significant reduction in circulating volume leads to decreased atrial filling, reduced cardiac output, and ultimately hypotension, unless sufficient interstitial fluid is available for intravascular refilling^25^. Although these factors are highly relevant for monitoring during hemodialysis treatment to enable appropriate interventions and improve patients’ quality of life, they are not, in themselves, predictive of the occurrence of falls.

Post-dialysis hypotension is associated with fall episodes, suggesting that hemodynamic instability in the post-session period is a critical factor to monitor^23^. In addition, excessive interdialytic weight gain—an indicator of fluid overload—was also associated with an increased risk of falls, possibly due to its contribution to balance disturbances and blood pressure fluctuations. One study reported that 32% of fall events in patients undergoing hemodialysis were related to weight variation during treatment, identifying this change as a significant risk factor. Excessive fluid removal is often associated with symptoms such as dizziness and weakness, which may precede falls^19^. These results corroborate the findings of the present study, in which 45.3% of participants reported post-dialysis hypotension and 51.5% had interdialytic weight gain greater than 4%. Consequently, 72.3% of fall events occurred at home or on streets after the hemodialysis session. Such events have previously been associated with falls, and the present study directly identifies weight fluctuations during HD sessions, fatigue, and intradialytic hypotension as factors directly related to the risk of falls.

The development of a fall risk assessment scale for hemodialysis patients represents a relevant strategy to strengthen patient safety actions in this high-risk group. The adoption of this instrument aims to provide a more comprehensive and accurate analysis of individual risk factors, considering clinical, functional, and environmental aspects that predispose patients to falls. The systematic application of the scale enabled early identification of the most vulnerable patients. It is expected to contribute significantly to the development of targeted and individualized preventive interventions, the standardization of care provided by the multidisciplinary team, the reduction in fall incidence, and the minimization of associated complications, ultimately improving the quality of life of patients undergoing dialysis therapy.

This study has as a limitation the application of the ERQUE_HD scale in only one dialysis center. Future studies should apply the scale in other centers with different patient profiles.

## Conclusions

The ERQUE_HD scale is predictive of fall risk, particularly among patients classified as being at high risk of this outcome. It is expected that the scale will assist in the implementation of preventive measures for this specific population, contributing to a reduction in the occurrence of this adverse event and promoting safer care for patients undergoing hemodialysis.

## Data Availability

The datasets generated and/or analyzed during the present study are available from the corresponding author upon reasonable request.

## References

[1] Hessels AJ, Paliwal M, Weaver SH, Siddiqui D, Wurmser TA (2019). Impact of patient safety culture on missed nursing care and
adverse patient events.. J Nurs Care Qual.

[2] World Health Organization. (2021). Global patient safety action plan 2021–2030: towards eliminating
avoidable harm in health care..

[3] Nerbass FB, Lima HN, Moura-Neto JA, Lugon JR, Sesso R (2024). Brazilian Dialysis Survey 2022.. Braz J Nephrol..

[4] Lessa SRO, Bezerra JNM, Barbosa SMC, Luz GOA, Borba AKOT (2018). Prevalência e fatores associados para a ocorrência de eventos
adversos no serviço de hemodiálise.. Texto Contexto Enferm.

[5] Sousa MRG, Silva AEBC, Bezerra ALQ (2016). Prevalência de eventos adversos em uma unidade de
hemodiálise.. Rev Enferm UERJ..

[6] Powell-Cope G, Quigley P, Besterman-Dahan K, Smith M, Stewart J, Melillo C, Haun J, Friedman Y (2014). A qualitative understanding of patient falls in inpatient mental
health units.. J Am Psychiatr Nurses Assoc..

[7] Zhao YL, Bott M, He J, Kim H, Park SH, Dunton N (2019). Evidence on fall and injurious fall prevention interventions in
acute care hospitals.. J Nurs Adm..

[8] Andrade D, Oliveira RA, Turrini RNT, Poveda VB (2019). Escalas de avaliação de risco para queda: revisão integrativa da
literatura.. Rev Baiana Enferm.

[9] Polit DF, Beck CT (2019). Fundamentos de pesquisa em enfermagem: avaliação de evidências para a
prática da enfermagem. 9. ed..

[10] Medeiros HP, Teixeira E (2016). Metodologia da pesquisa para a enfermagem e saúde: resenha de
livro.. Rev Bras Enferm.

[11] Souza MT, Silva MD, Carvalho R (2010). Revisão integrativa: o que é e como fazer.. Einstein (Sao Paulo).

[12] Mendes KDS, Silveira RCCP, Galvão CM (2019). Use of the bibliographic reference manager in the selection of
primary studies in integrative reviews.. Texto Contexto Enferm.

[13] Alexandre NMC, Coluci MZO (2011). Validade de conteúdo nos processos de construção e adaptação de
instrumentos de medidas.. Ciênc saúde coletiva..

[14] Rubio DM, Berg-Weger M, Tebb SS, Lee ES, Rauch S (2003). Objectifying content validity: conducting a content validity
study in social work research.. Soc Work Res.

[15] Hosmer DW, Lemeshow S (2013). Sturdivant RX. Applied logistic regression..

[16] Grant JS, Davis LL (1997). Selection and use of content experts for instrument
development.. Res Nurs Health.

[17] World Health Organization (WHO) (2015). Number of people over 60 years set to double by 2050; major societal
changes required.

[18] Dias AL, Pereira FA, Barbosa CP, Araújo-Monteiro GK, Santos-Rodrigues RC, Souto RQ (2023). Risco de quedas e a síndrome da fragilidade no
idoso.. Acta Paul Enferm.

[19] de Jesus LAS, Lucinda LMF, Cobucci RF, de Oliveira HB, Rangel PRB, de Oliveira BCB (2021). Quedas em pacientes em hemodiálise: um estudo piloto prospectivo
de 12 meses.. HU Rev..

[20] Nazário SS, Cruz EDA, Batista J, Silva DP, Pedro RL, Laynes RL (2022). Caracterização de eventos adversos hospitalares: busca ativa
versus notificação espontânea.. Cogitare Enferm.

[21] da Silva TP, Venancio JB, Oliveira MJ, Lima AC, Santos BMP, Ferreira BM (2022). A influência da utilização de medicamentos no risco de quedas em
idosos de instituições de longa permanência do Distrito
Federal.. Braz J Dev..

[22] Antonio GAF, Ribeiro A, Ribeiro AM, Fernandes BAM, Dourado C, Torres FCV (2025). Neuropatia diabética: conhecer para reconhecer..

[23] Carvalho TC, Dini AP (2020). Risk of falls in people with chronic kidney disease and related
factors.. Rev Lat Am Enfermagem.

[24] Daugirdas JT, Blake PG, Ing TS (2017). Manual de diálise..

[25] Meira FS, Figueiredo AE, Zemiarcki J, Pacheco J, Poli-de-Figueiredo CE, d’Avila DO (2010). Two variable sodium profiles and adverse effects during
hemodialysis: a randomized crossover study.. Ther Apher Dial.

[26] McAdams-DeMarco MA, Suresh S, Law A, Salter ML, Gimenez LF, Jaar BG (2013). Frailty and falls among adult patients undergoing chronic
hemodialysis: a prospective cohort study.. BMC Nephrol.

[27] Delgado C, Shieh S, Grimes B, Chertow GM, Dalrymple LS, Kaysen GA (2015). Association of self-reported frailty with falls and fractures
among patients new to dialysis.. Am J Nephrol.

[28] Erken E, Ozelsancak R, Sahin S, Yılmaz EE, Torun D, Leblebici B (2016). The effect of hemodialysis on balance measurements and risk of
fall.. Int Urol Nephrol.

[29] Bowling CB, Bromfield SG, Colantonio LD, Gutiérrez OM, Shimbo D, Reynolds K (2016). Association of Reduced eGFR and Albuminuria with Serious Fall
Injuries among Older Adults.. Clin J Am Soc Nephrol.

[30] Kono K, Nishida Y, Yabe H, Moriyama Y, Mori T, Shiraki R (2018). Development and validation of a Fall Risk Assessment Index for
dialysis patients.. Clin Exp Nephrol.

[31] Plantinga LC, Patzer RE, Franch HA, Bowling CB (2017). Serious fall injuries before and after initiation of hemodialysis
among older ESRD Patients in the United States: a retrospective Cohort
Study.. Am J Kidney Dis.

[32] van Loon IN, Joosten H, Iyasere O, Johansson L, Hamaker ME, Brown EA (2019). The prevalence and impact of falls in elderly dialysis patients:
Frail elderly Patient Outcomes on Dialysis (FEPOD) study.. Arch Gerontol Geriatr.

[33] Wang HH, Wu JL, Lee YC, Ho LC, Chang MY, Liou HH (2020). Risk of serious falls between hemodialysis and peritoneal
dialysis patients: a nationwide population-based Cohort
Study.. Sci Rep.

[34] Zanotto T, Mercer TH, van der Linden ML, Rush R, Traynor JP, Petrie CJ (2020). The relative importance of frailty, physical and cardiovascular
function as exercise-modifiable predictors of falls in haemodialysis
patients: a prospective cohort study.. BMC Nephrol.

[35] Vanden Wyngaert K, Celie B, Calders P, Eloot S, Holvoet E, Van Biesen W (2020). Markers of protein-energy wasting and physical performance in
haemodialysis patients: A cross-sectional study.. PLoS One.

[36] Perez-Gurbindo I, María Álvarez-Méndez A, Pérez-García R, Cobo PA, Carrere MTA (2021). Factors associated with falls in hemodialysis patients: a
case-control study.. Rev Lat Am Enfermagem.

[37] Shirai N, Yamamoto S, Osawa Y, Tsubaki A, Morishita S, Narita I (2022). Dysfunction in dynamic, but not static balance is associated with
risk of accidental falls in hemodialysis patients: a prospective cohort
study.. BMC Nephrol.

[38] Shirai N, Inoue T, Ogawa M, Okamura M, Morishita S, Suguru Y (2022). Relationship between nutrition-related problems and falls in
hemodialysis patients: a narrative review.. Nutrients.

[39] Liu X, Chen S, Liu C, Dang X, Wei M, Xin X (2023). Novel risk-factor analysis and risk-evaluation model of falls in
patients receiving maintenance hemodialysis.. Ren Fail.

[40] Liang J, Wang Y, Zhang W, Ding H, Gao Y, Wang R (2023). Incidence and risk factors of falls in patients undergoing
hemodialysis: a multicenter survey in northern China.. Hemodial Int.

[41] Ishii T, Matsumoto W, Hoshino Y, Kagawa Y, Iwasaki E, Takada H (2023). Walking aids and complicated orthopedic diseases are risk factors
for falls in hemodialysis patients: an observational study.. BMC Geriatr.

