# Supplementary material for: Fall risk assessment scale for hemodialysis patients (ERQUE_HD): development and validation
Source: J Bras Nefrol. 2026 Jun 5;48(3):e20250283. doi: 10.1590/2175-8239-JBN-2025-0283en (PMC13248899; doi:10.1590/2175-8239-JBN-2025-0283en)
Supplement: Translation

# Translation: Escala de avaliação do risco de quedas em pacientes na hemodiálise (ERQUE_HD): desenvolvimento e validação

**DOI:** 10.1590/2175-8239-JBN-2025-0283pt

**Authors:** Veronica Alacarini Farina, Simone Travi Canabarro, Gabriela de Souza, Lucas Teixeira Negrello, Ana Elizabeth Prado Lima Figueiredo

**Affiliations:** 1 Universidade Federal de Ciências da Saúde de Porto Alegre, Porto Alegre, RS, Brasil.; 2 Pontifícia Universidade Católica do Rio Grande do Sul, Porto Alegre, RS, Brasil.

**Keywords:** Acidentes por Quedas, Segurança do Paciente, Diálise Renal, Fatores de Risco, Enfermagem

## Abstract

**Introdução:**

Pacientes em hemodiálise apresentam múltiplas comorbidades e complicações durante a terapia, fatores que aumentam o risco de quedas. A avaliação adequada desse risco é fundamental para a implementação de medidas preventivas eficazes.

**Objetivo:**

Desenvolver e validar uma escala para avaliação do risco de quedas para pacientes em hemodiálise.

**Método:**

Trata-se de uma pesquisa metodológica de caráter quantitativo e qualitativo, realizada em cinco etapas: revisão integrativa, realização de grupos focais, construção do instrumento, validação do conteúdo e validação da escala.

**Resultados:**

A revisão integrativa incluiu 17 artigos e subsidiou a elaboração inicial dos itens. Nove grupos focais, com pacientes e profissionais de saúde, permitiram identificar novos fatores de risco, resultando na construção da ERQUE_HD (Escala de Avaliação do Risco de Quedas em Pacientes na Hemodiálise), composta por 17 itens distribuídos em quatro domínios: histórico de saúde, fatores dialíticos, capacidade funcional e nutrição. A validação do conteúdo obteve um índice de validade de conteúdo (IVC) de 0,95, valor considerado significativo. Por fim, a aplicação foi realizada em 66 pacientes, e a análise foi feita por regressão logística com equações de estimação generalizadas (EEG), incluindo variáveis como fadiga pós-diálise, ganho de peso interdialítico e hipotensão intradialítica. Aqueles com risco elevado apresentaram probabilidade 8,7 vezes maior de queda em relação ao grupo de risco moderado. A ERQUE_HD demonstrou boa acurácia (AUC = 0,817). Fadiga pós- diálise e ganho de peso >4% entre sessões foram preditores relevantes de queda.

**Conclusão:**

A ERQUE_HD mostrou-se eficaz na avaliação e predição do risco de quedas em pacientes em hemodiálise.

## Introdução

As exigências associadas à segurança e à qualidade assistencial vêm crescendo e se disseminando mundialmente nas últimas décadas, gerando esforços significativos para melhorar a cultura de segurança do paciente em instituições de saúde^1^. De acordo com o Plano de Ação Global, uma nova definição para a segurança do paciente foi estabelecida: um conjunto estruturado de atividades que promove culturas, processos, procedimentos, comportamentos, tecnologias e ambientes na assistência à saúde, os quais reduzem riscos de forma consistente e sustentável, minimizam a ocorrência de danos evitáveis, tornam os erros menos prováveis e reduzem o impacto dos danos quando eles ocorrem^2^.

Um dos protocolos de segurança do paciente é a prevenção dos eventos de queda, que podem ocorrer tanto em pacientes internados em instituições hospitalares quanto naqueles em tratamento ambulatorial. Indivíduos com doença renal crônica (DRC) frequentemente necessitam de diálise, sendo a hemodiálise (HD) uma das modalidades mais utilizadas. Estudos anuais realizados em centros brasileiros de diálise apontam que, em julho de 2023, o número total de pacientes em diálise era de 157.357, dos quais 51.153 iniciaram a terapia no mesmo ano^3^.

A HD pode ser realizada em ambiente hospitalar ou em clínicas, de forma ambulatorial, nas quais o paciente realiza a terapia e é liberado após o procedimento. Trata-se de uma terapia de alta complexidade, devido à necessidade de equipamentos específicos, à realização de procedimentos invasivos para obtenção do acesso vascular e ao uso de medicamentos, como anticoagulantes, durante as sessões^4^.

Diante desse cenário, torna-se fundamental mitigar os riscos dos possíveis eventos adversos (EAs) que possam ocorrer com o paciente em HD, como reduções abruptas da pressão arterial, infecções de acesso vascular, erros de medicação e o evento de quedas. Frequentemente, os pacientes que necessitam dessa terapia apresentam condições clínicas desfavoráveis, além de possuírem diversas comorbidades, e qualquer tipo de falha que venha a ocorrer pode trazer consequências graves à saúde^5^.

Apesar de se reconhecer que não é possível eliminar o risco de quedas, uma avaliação assertiva, considerando as individualidades e os diferentes perfis, pode minimizar consideravelmente esse risco e permitir a implementação de medidas individualizadas para cada paciente^6,7^. É de extrema importância a avaliação por meio do uso de uma escala validada; atualmente, as ferramentas existentes que mensuram esse risco foram desenvolvidas na década de 1990 e têm como foco, principalmente, pacientes clínicos, geriátricos e cirúrgicos hospitalizados^8^.

Entretanto, ainda não existem escalas validadas capazes de apoiar adequadamente a estratificação do risco de quedas em pacientes adultos em terapia hemodialítica, evidenciando uma lacuna importante na assistência direcionada a esse público.

O presente estudo teve como objetivo a construção e validação de uma escala para avaliar o risco de quedas em pacientes em hemodiálise.

## Método

Trata-se de um estudo caracterizado como pesquisa metodológica, com abordagem quantitativa e qualitativa, para a construção e validação da escala denominada Escala de Avaliação do Risco de Quedas em Pacientes na Hemodiálise (ERQUE_HD). Estudos de caráter metodológico são capazes de subsidiar a construção de conhecimento a partir da busca pela melhor forma de medir o fenômeno, seja por meio do desenvolvimento, validação e avaliação de ferramentas e métodos de pesquisa. Em geral, são de caráter não experimental e concentram-se na criação de novos instrumentos^9,10^. O projeto foi aprovado pelo Comitê de Ética em Pesquisa, sob o CAAE nº 65446322.5.0000.5345, conforme exigido pelas normas da Plataforma Brasil. O estudo foi realizado de acordo com as etapas descritas na Figura 1.

**Figura 1:** Etapas do desenvolvimento da Escala ERQUE_HD.

Cada etapa foi desenvolvida conforme descrito abaixo:

1. **Revisão da literatura:** As etapas utilizadas foram: 1) elaboração da pergunta norteadora da revisão; 2) busca e seleção dos estudos primários na literatura; 3) extração de dados dos estudos; 4) avaliação crítica dos estudos primários incluídos na revisão; 5) síntese dos resultados da revisão; e 6) apresentação do método^11^. A questão norteadora foi elaborada com a estratégia PICO: “Quais são os principais fatores de risco de quedas em pacientes em hemodiálise?”^12^. A Figura 2 mostra, com mais detalhes, a realização desta revisão.
2. **Grupos focais:** Foram realizados nove grupos focais, envolvendo pacientes, técnicos de enfermagem dos dois centros participantes e especialistas de diferentes instituições de saúde. Os critérios de inclusão para os especialistas foram: titulação mínima de mestre em áreas relacionadas à nefrologia ou à segurança do paciente e experiência profissional superior a cinco anos na área de atuação, incluindo assistência a pacientes em HD, gestão de risco e qualidade e/ou segurança do paciente. Esses grupos focais foram compostos por médicos, enfermeiros, nutricionistas, educadores físicos e fisioterapeutas. Já os participantes do grupo focal de pacientes foram selecionados por conveniência, contemplando diferentes faixas etárias, todos com idade igual ou superior a 18 anos. Do total de 16 participantes, 11 eram do sexo masculino e cinco do sexo feminino, todos em terapia hemodialítica há, no mínimo, dois anos e distribuídos em diferentes turnos de tratamento, o que permitiu a inclusão de distintos perfis e estilos de vida. Para todos os grupos, os critérios de exclusão adotados foram a recusa em participar do estudo ou a presença de comprometimentos que impossibilitassem a compreensão das perguntas e a participação nas discussões. Os grupos focais foram realizados no período de abril de 2023 a maio de 2024. Todos os participantes elegíveis assinaram o Termo de Consentimento Livre e Esclarecido (TCLE) de forma presencial. Os encontros ocorreram presencialmente, com duração média de 60 minutos, e todas as sessões foram gravadas em áudio para garantir a fidedignidade dos dados, após o que foram transcritas e lidas pelo pesquisador responsável. A interação presencial favoreceu a troca de experiências e a obtenção de *insights* mais aprofundados.
3. **Organização dos itens:** Os itens identificados na revisão foram organizados em uma tabela e validados por meio da análise das transcrições dos grupos focais, com base nas experiências relatadas. Em seguida, foram agrupados em quatro domínios temáticos: histórico de saúde, fatores dialíticos, capacidade funcional e nutrição. A primeira versão da escala, com 19 itens, foi então elaborada. Posteriormente, um novo grupo focal com especialistas avaliou a aplicabilidade de cada item, resultando em ajustes e na finalização da segunda versão da escala.
4. **Validação do conteúdo:** A etapa foi composta por um comitê de 15 especialistas em segurança do paciente, de diversas instituições de saúde, dentre médicos, farmacêuticos e enfermeiros que possuíam mestrado e/ou doutorado, selecionados e convidados por conveniência, os quais validaram o conteúdo de maneira virtual. Todos receberam um e-mail convidando-os a participar, com o TCLE em anexo para assinatura. Para validação dos itens da escala, estes foram pontuados em escala *Likert*, permitindo avaliar item por item, além de um campo para a escrita de sugestões de melhoria ou comentários^13,14^.
5. **Teste da escala:** Na etapa de validação, a escala foi aplicada a pacientes de diferentes faixas etárias e em diferentes turnos de um centro participante, abrangendo os três períodos (manhã, tarde e noite), uma vez por semana a cada participante, de forma consecutiva por quatro semanas. Após essa aplicação, os resultados foram organizados em uma tabela e analisados estatisticamente. É importante ressaltar que o item referente a quedas no último ano foi adaptado para quedas na última semana, devido a alguns pacientes não estarem em tratamento há um ano, o que possibilitou uma melhor aplicabilidade do item, bem como da escala, que passou a ser empregada semanalmente, permitindo identificar quedas desde a última aplicação e mudanças no escore. O tratamento estatístico dos dados foi realizado com o auxílio do programa estatístico *Statistical Package for Social Sciences*, versão 25.0 (SPSS Inc., Chicago, IL, EUA, 2018), para Windows. A apresentação dos resultados ocorreu por meio de estatística descritiva, envolvendo as distribuições absoluta e relativa (n, %), bem como as medidas de tendência central (média e mediana) e de variabilidade (desvio padrão e amplitude interquartil), com estudo da simetria das distribuições contínuas, analisada pelo teste de *Kolmogorov-Smirnov*. A comparação das variáveis categóricas entre as semanas foi realizada por meio do teste de *Friedman*. Quanto à comparação entre variáveis categóricas e classificações de risco e desfechos de queda, foi empregado o teste exato de Fisher (simulações de Monte Carlo). Para o estudo da predição da ocorrência de queda no último ano em função da classificação de risco, bem como em função de outras covariáveis relevantes, ao longo do seguimento, foi aplicada a técnica de regressão de *Poisson* com variância robusta, por meio de Estimativas de Equações Generalizadas (EEG). A análise dos preditores, apresentada pelo *odds ratio* (OR), foi realizada inicialmente de forma univariada, e as variáveis com valor de p <0,05 foram consideradas para compor o modelo multivariado. Já a curva *Receiver Operating Characteristic* (ROC) foi construída utilizando as pontuações da escala ERQUE_HD para discriminar os participantes com e sem ocorrências de queda no último ano, segundo a classificação estimada pelo modelo preditivo. A área sob a curva (AUC) foi descrita com intervalo de confiança de 95%. Hosmer e Lemeshow^15^ sugerem que uma AUC entre 0,70 e 0,80 é “aceitável”, entre 0,80 e 0,90 é “excelente” e acima de 0,90 é “excepcional”. Foram calculados, ainda, os índices de *Youden* (sensibilidade + especificidade − 1) para determinar o valor da escala ERQUE_HD com melhor desempenho discriminatório.

**Figura 2:** Revisão integrativa.

## Resultados

Os resultados serão descritos de acordo com as etapas realizadas, na mesma ordem em que foram apresentadas na seção Métodos.

1. **Realização da revisão integrativa:** Os artigos identificados foram importados para a plataforma **Rayyan** para triagem. Inicialmente, 94 estudos foram encontrados nas bases Medline/PubMed, **Web of Science**, SciELO, EBSCO, CINAHL e BVS. Após a exclusão de 27 duplicatas, restaram 77 artigos, dos quais 33 foram eliminados após a leitura dos títulos e resumos. Dos 44 selecionados, 29 foram avaliados na íntegra, resultando na inclusão final de 17 artigos que preencheram os critérios da revisão, os quais foram lidos na íntegra e organizados em uma planilha com os principais fatores de risco, descritos na Tabela 1.
2. **Realização dos grupos focais:** Foi realizada uma leitura minuciosa de todas as transcrições dos grupos focais e os fatores de risco mencionados pelos participantes foram identificados e categorizados em formato de planilha, facilitando sua visualização e análise comparativa. Os fatores relevantes identificados foram: depressão, ansiedade, idade, um dos grupos citou o sexo feminino, e seis grupos citaram o sexo masculino como fator de risco, deficiência visual, deficiência auditiva, fadiga, hipoglicemia, auxílio à marcha, hipotensão postural, cãibras durante a sessão, peso interdialítico, falta da prática de exercício, doenças ósseas, uso de medicamentos (anti-hipertensivos), fraqueza muscular, desequilíbrio do eixo postural, capacidade funcional, diabetes, hipertensão e histórico de quedas.
3. **Construção do instrumento:** Compilando os itens da revisão integrativa e os fatores abordados em todos os grupos focais, os de maior relevância foram agrupados em domínios específicos e ajustados conforme cada discussão. No geral, foram contemplados 17 itens, divididos em quatro domínios, sendo eles: histórico de saúde, fatores dialíticos, capacidade funcional e nutrição.
4. **Validação do conteúdo:** Para esta etapa, foi apurado o índice de validade de conteúdo (IVC), cujo escore foi calculado a partir da soma das pontuações atribuídas a cada item pelos especialistas, considerando-se aquelas classificadas como “3” ou “4”^16^. Os itens com pontuação “1” ou “2” precisaram ser revisados ou excluídos, por terem sido classificados como “1: não relevante” ou “2: necessita de revisão para avaliação da relevância”. A escala foi finalizada, sendo sua versão final apresentada na Figura 3, com um total de 17 itens. Considerando os IVC dos itens mantidos, a média foi de 0,95, o que reforça que o conteúdo da escala foi validado de acordo com os valores recomendados. Conforme demonstrado na Tabela 2, dos 19 itens iniciais, dois foram retirados da escala por apresentarem valor inferior ao recomendado, sendo eles sexo e apetite, os quais não foram considerados relevantes.
5. **Validação da escala:** A escala foi aplicada em um dos centros participantes durante quatro semanas, nos três turnos de funcionamento. Os resultados referem-se a uma amostra inicial de 66 pacientes acompanhados ao longo desse período. Nas semana um (S1) e semana dois (S2), o número total de participantes foi mantido, embora três pacientes tenham deixado o seguimento por óbito, mudança de estado ou internação, sendo substituídos por outros três. Na semana três (S3), observou-se uma redução de 9,1% (n = 6) na amostra, restando 60 pacientes, e na semana quatro (S4), houve nova redução de 10,6% (n = 7), totalizando 59 participantes ao final do seguimento.

**Tabela 1:** Quadro sinóptico dos artigos selecionados, com os principais fatores de risco para quedas

Título	Autores	País/Ano	Metodologia	Objetivos	Conclusão/Principais fatores que levam a quedas
Frailty and falls among adult patients undergoing chronic hemodialysis: a prospective cohort study^26^	Mara A McAdams-DeMarco; Sunitha Suresh; Andrew Law; Megan L Salter; Luis F Gimenez; Bernard G Jaar; Jeremy D Walston; Dorry L Segev.	Estados Unidos, 2013	Estudo longitudinal	Identificar se a fragilidade estava associada a quedas, independentemente de outros fatores de risco, em pacientes em hemodiálise pertencentes a todas as faixas etárias.	Fragilidade; idade avançada; comorbidades; uso de medicamentos.
Association of Self-Reported Frailty with Falls and Fractures among Patients New to Dialysis^27^	Cynthia Delgado; Stephanie Shieh; Barbara Grimes; Glenn M. Chertow; Lorien S. Dalrymple; George A. Kaysen; John Kornak; and Kirsten L. Johansen.	Estados Unidos, 2015	Estudo de coorte prospectivo	Examinar as taxas de quedas ou fraturas que requerem consulta médica ou hospitalização em uma coorte diversificada de pacientes inscritos no Comprehensive Dialysis Study, um estudo especial do U.S. Renal Data System.	Fragilidade; histórico de quedas.
The effect of hemodialysis on balance measurements and risk of fall^28^	Erken E; Ozelsancak R; Sahin S; Yılmaz EE; Torun D; Leblebici B; Kuyucu YE; Sezer S.	Turquia, 2016	Estudo caso-controle	Investigar o efeito do tratamento de hemodiálise no equilíbrio e avaliar a possível relação entre a hemodiálise e o aumento da incidência de lesões por queda em pacientes com DRT.	Fadiga; idade; baixa hemoglobina; hipotensão; polifarmácia; diálise inadequada; distúrbios eletrolíticos.
Association of Reduced eGFR and Albuminuria with Serious Fall Injuries among Older Adults^29^	C Barrett Bowling; Samantha G Bromfield; Lisandro D Colantonio; Orlando M Gutiérrez; Daichi Shimbo; Kristi Reynolds; Nicole C Wright; Jeffrey R Curtis; Suzanne E Judd; Harold Franch; David G Warnock; William McClellan; Paul Muntner.	Estados Unidos, 2016	Estudo de coorte prospectivo	Determinar a associação entre TFGe reduzida e relação albumina-creatinina (RAC) elevada e o risco de lesões graves por queda, definidas como fratura, lesão cerebral ou luxação articular relacionada à queda.	Taxas reduzidas de TFGe.
Development and validation of a Fall Risk Assessment Index for dialysis patients^30^	Kono K; Nishida Y; Yabe H; Moriyama Y; Mori T; Shiraki R; Sato T.	Japão, 2017	Estudo de coorte epidemiológica	Desenvolver um formulário abrangente de avaliação de sintomas para prever quedas com alta sensibilidade em pacientes em diálise.	Idade avançada; estado inflamatório; desnutrição; hipotensão; equilíbrio; histórico de quedas.
Serious Fall Injuries Before and After Initiation of Hemodialysis Among Older ESRD Patients in the United States: A Retrospective Cohort Study^31^	Plantinga LC; Patzer RE; Franch HA; Bowling CB.	Estados Unidos, 2017	Estudo de coorte retrospectivo	Determinar a taxa de lesões graves por quedas entre pacientes idosos em hemodiálise e verificar se essa taxa difere antes e depois do início da terapia dialítica.	Idosos; período pós-HD comparado ao pré.
The prevalence and impact of falls in elderly dialysis patients: Frail Elderly Patient Outcomes on Dialysis (FEPOD) study^32^	Ismay N. van Loon; Hanneke Joosten; Osasuyi Iyasere; Lina Johansson; Marije E. Hamaker; Edwina A. Brown.	Reino Unido, 2019	Estudo longitudinal observacional	Avaliar a prevalência de quedas e fraturas em idosos frágeis em diálise, bem como sua relação com mortalidade e hospitalização, além da influência das quedas no desempenho funcional e na qualidade de vida (QV).	Fragilidade; diabetes; qualidade de vida; idade avançada.
Risk of falls in people with chronic kidney disease and related factors^23^	Thaís Carrera de Carvalho; Ariane Polidoro Dini.	Brasil, 2020	Estudo quantitativo, descritivo e de associação	Identificar o risco e a prevalência de quedas no último ano em doentes renais crônicos em hemodiálise; associar o risco de queda ao medo de cair e a variáveis sociodemográficas.	Diabéticos; auxílio para deambulação; uso de órteses.
Risk of Serious Falls Between Hemodialysis and Peritoneal Dialysis Patients: A Nationwide Population-based Cohort Study^33^	Wang HH; Wu JL; Lee YC; Ho LC; Chang MY; Liou HH; Hung SY.	China, 2020	Estudo de coorte	Estimar o risco de quedas graves entre pacientes em HD e DP, a fim de determinar qual modalidade de diálise está mais associada a esse desfecho.	Idade avançada; problemas cardiovasculares; histórico de quedas antes da diálise; mulheres; acidente vascular cerebral; alterações físicas, sensoriais e cognitivas relacionadas ao envelhecimento; diuréticos, bloqueadores e midriáticos; fragilidade; fadiga pós-diálise; desnutrição; velocidade da marcha.
The relative importance of frailty, physical and cardiovascular function as exercise-modifiable predictors of falls in haemodialysis patients: a prospective cohort study^34^	Zanotto T; Mercer TH; van der Linden ML; Rush R; Traynor JP; Petrie CJ; Doyle A; Chalmers K; Allan N; Shilliday I; Koufaki P.	Reino Unido, 2020	Estudo prospectivo observacional	Explorar a importância relativa da fragilidade e da função cardiovascular como potenciais preditores de quedas modificáveis pelo exercício em pacientes com DRC-5 em HD.	Fragilidade; força de preensão manual; número de passos diários; velocidade da marcha; TUG; função cardiovascular; hipotensão; baixa frequência de ativação do barorreflexo (BEI); anti-hipertensivos.
Markers of protein-energy wasting and physical performance in haemodialysis patients: A cross-sectional study^35^	Vanden Wyngaert K; Celie B; Calders P; Eloot S; Holvoet E; Van Biesen W; Van Craenenbroeck AH.	Bélgica, 2020	Estudo transversal	Avaliar a relação transversal entre estado nutricional, força muscular, capacidade de exercício e risco de quedas.	Estado nutricional; fragilidade; força muscular de membros inferiores.
Factors associated with falls in hemodialysis patients: a case-control study^36^	Ignacio Perez-Gurbindo; Ana Maria Álvarez-Méndez; Rafael Pérez García; Patrícia Arribas-Cobo; Maria Teresa Angulo-Carrére.	Espanha, 2021	Estudo caso-controle	Identificar possíveis associações entre maior probabilidade de quedas em pacientes em hemodiálise e valores laboratoriais, comorbidades, tratamento farmacológico, alterações hemodinâmicas, desfechos da diálise e alterações estabilométricas.	Peso intradialítico (>1,9kg); pacientes com resposta cronotrópica limitada por causas intrínsecas ou extrínsecas correm maior risco de quedas quando submetidos à HD; anti-hipertensivos; níveis mais elevados de β2-microglobulina.
Dysfunction in dynamic, but not static balance is associated with risk of accidental falls in hemodialysis patients: a prospective cohort study^37^	NobuyukiShirai; Suguru Yamamoto; Yutaka Osawa; Atsuhiro Tsubaki; Shinichiro Morishita; Ichiei Narita.	Japão, 2022	Estudo observacional prospectivo	Avaliar os detalhes das quedas acidentais e sua associação com a função de equilíbrio em pacientes em HD.	Idade avançada; associação do teste Timed Up and Go (TUG); homens idosos ativos com baixa função física, mais propensos; equilíbrio dinâmico.
Relationship between Nutrition-Related Problems and Falls in Hemodialysis Patients: A Narrative Review^38^	Shirai N; Inoue T; Ogawa M; Okamura M; Morishita S; Suguru Y; Tsubaki A.	Japão, 2022	Revisão narrativa	Resumir o impacto da fragilidade, sarcopenia, desnutrição, protein-energy wasting (PEW) e caquexia nas quedas em pacientes em HD.	Fragilidade; desnutrição; atrofia muscular; equilíbrio postural; hipotensão; retirada de líquidos; idade; sexo masculino; histórico de quedas; uso de antidepressivos; polifarmácia; baixa qualidade de vida; força muscular de membros inferiores e função física; diabetes; paratormônio elevado, PCR elevado.
Novel risk-factor analysis and risk-evaluation model of falls in patients receiving maintenance hemodialysis^39^	Liu X; Chen S; Liu C; Dang X; Wei M; Xin X; Gao J.	China, 2022	Estudo transversal	Investigar a prevalência de quedas em pacientes com DRC, analisar fatores de risco para quedas e construir um modelo de avaliação de risco.	Idade; deficiência auditiva; uso de auxiliares de marcha; ansiedade; depressão; fraqueza.
Incidence and risk factors of falls in patients undergoing hemodialysis: A multicenter survey in northern China^40^	Liang J; Wang Y; Zhang W; Ding H; Gao Y; Wang R; Sun X; Peng Y; Gan L; Zuo L.	China, 2023	Estudo prospectivo e longitudinal	Fornecer um esboço claro da incidência de quedas no norte da China e identificar fatores de risco associados em pacientes de todas as idades em hemodiálise.	Idade avançada (associada à fragilidade); anemia (associada à fadiga, fraqueza e baixa energia); anormalidades na marcha e na função.
Walking aids and complicated orthopedic diseases are risk factors for falls in hemodialysis patients: an observational study^41^	Ishii T; Matsumoto W; Hoshino Y; Kagawa Y; Iwasaki E; Takada H; Honma T; Oyama K.	Japão, 2023	Estudo observacional	Explorar estatisticamente os fatores relacionados às quedas acidentais em instalações de diálise para futura prevenção de eventos.	Auxílio de marcha; histórico de quedas; deficiência visual; incontinência urinária; limitação funcional; idosos; uso auxiliar de marcha.

**Figura 3:** Escala ERQUE_HD.

**Tabela 2:** Itens da escala conforme o índice de validação do conteúdo

Item	IVC	Classificação na escala
1. Sexo	0,53	Item retirado
2. Idade	1,00	Item mantido
3. Deficiência visual e auditiva	1,00	Item mantido
4. Limitação motora	1,00	Item mantido
5. Uso de medicamentos	1,00	Item mantido
6. Histórico de quedas	1,00	Item mantido
7. Diabetes mellitus	0,86	Item mantido
8. Doença óssea diagnosticada	0,86	Item mantido
9. Fadiga pós-diálise na última semana	0,93	Item mantido
10. Hipotensão intradialítica	1,00	Item mantido
11. Hipotensão pós-diálise	1,00	Item mantido
12. Outras complicações intradialíticas	0,93	Item mantido
13. Uso de dispositivos para auxílio na marcha	1,00	Item mantido
14. Marcha	1,00	Item mantido
15. Força	1,00	Item mantido
16. Realiza algum tipo de atividade física organizada?	0,86	Item mantido
17. Índice de massa corporal	0,93	Item mantido
18. Apetite	0,60	Item retirado
19. Ganho de peso entre as sessões superior a 4%	0,86	Item mantido
Soma dos itens mantidos (IVC):	16,23/17 = 0,95	

A Tabela 3 apresenta a caracterização da amostra em cada semana, considerando as variações no número e na composição dos pacientes acompanhados. De acordo com os resultados, não foram detectadas diferenças estatisticamente significativas na comparação das variáveis investigadas em relação às semanas. Dessa forma, embora tenham ocorrido variações no número de pacientes, bem como nas respostas apontadas, estas não se mostraram relevantes, indicando que as semanas apresentaram características semelhantes em relação às variáveis investigadas.

**Tabela 3:** Distribuição absoluta e relativa das características gerais da amostra por semana do seguimento

Características da amostra	Semanas^a^								p^b^
	Semana 1 – S1 (n = 66)		Semana 2 – S2 (n = 66)		Semana 3 – S3 (n = 60)		Semana 4 – S4 (n = 59)		
	n	%	n	%	n	%	n	%	
Idade									0,516
Entre 60 e 80 anos (2)	39	59,1	34	51,5	36	60,0	31	52,5	
> 80 anos (3)	7	10,6	9	13,6	6	10,0	9	15,3	
< 60 anos (1)	20	30,3	23	34,8	18	30,0	19	32,2	
Deficiência visual ou auditiva									0,122
Não (0)	27	40,9	33	50,0	37	61,7	21	35,6	
Sim, ambas (3)	7	10,6	7	10,6	5	8,3	3	5,1	
Sim, auditiva (1)	4	6,1	2	3,0	1	1,7	1	1,7	
Sim, visual (2)	28	42,4	24	36,4	17	28,3	34	57,6	
Limitação motora									0,711
Ambas			2	3,0					
Membros inferiores (2)	18	27,3	17	25,8	12	20,0	12	20,3	
Membros superiores (1)	1	1,5							
Não (0)	47	71,2	47	71,2	48	80,0	47	79,7	
Uso de medicamentos									0,822
Não utiliza (0)	3	4,5			2	3,3			
Sim, utiliza 1 medicamento (1)	1	1,5	1	1,5	1	1,7			
Sim, utiliza 2 medicamentos (2)	62	93,9	65	98,5	57	95,0	59	100,0	
Diabetes mellitus									0,942
Não (0)	39	59,1	42	63,6	36	60,0	36	61,0	
Sim (1)	27	40,9	24	36,4	24	40,0	23	39,0	
Doença óssea									0,879
Não (0)	60	90,9	61	92,4	55	91,7	56	94,9	
Sim (1)	6	9,1	5	7,6	5	8,3	3	5,1	
Fadiga pós-diálise: “Sentiu-se cansado após as sessões de hemodiálise?” “A fadiga limitou ou atrapalhou suas atividades diárias nos dias subsequentes?”									0,758
Não para as 2 questões (0)	17	25,8	18	27,3	14	23,3	17	28,8	
Sim para uma questão (1)	17	25,8	19	28,8	21	35,0	17	28,8	
Sim para as duas questões (2)	32	48,5	29	43,9	25	41,7	25	42,4	
Hipotensão intradialítica									0,073
Não (0)	27	40,9	32	48,5	35	58,3	37	62,7	
Sim (1)	39	59,1	34	51,5	25	41,7	22	37,3	
Hipotensão pós-dialise									0,376
Não (1)	36	54,5	30	45,5	36	60,0	39	66,1	
Sim, esperou no mínimo 30 minutos para ir embora (2)	28	42,4	29	43,9	22	36,7	18	30,5	
Sim, não esperou 30 minutos para ir embora (3)	2	3,0	7	10,6	2	3,3	2	3,4	
Outras complicações (cãibras, dor lombar, febre, calafrios e hipoglicemia)									0,206
Não (1)	15	22,7	21	31,8	18	30,0	19	32,2	
Sim, duas ou mais complicações (3)	18	27,3	12	18,2	4	6,7	4	6,8	
Sim, uma complicação (2)	33	50,0	33	50,0	38	63,3	36	61,0	
Uso de dispositivo para auxílio na marcha									
Não utiliza/Acamado (0)	50	75,8	49	74,2	48	80,0	48	81,4	0,447
Sim, apenas para deslocamento fora do domicílio (1)	3	4,5	3	4,5	1	1,7	1	1,7	
Sim, sempre, no domicílio e fora dele (2)	13	19,7	14	21,2	11	18,3	10	16,9	
Marcha									0,156
Marcha comprometida (2)	14	21,2	18	27,3	11	18,3	17	28,8	
Marcha normal (1)	49	74,2	46	69,7	45	75,0	40	67,8	
Não deambula (0)	3	4,5	2	3,0	4	6,7	2	3,4	
Força, ficar na ponta dos pés									0,167
Não consegue realizar (2)	21	31,8	26	39,4	15	25,0	19	32,2	
Sim, consegue realizar em amplitude (1)	45	68,2	40	60,6	45	75,0	40	67,8	
Índice de massa corporal									0,142
Adequado (1)	25	37,9	21	31,8	25	41,7	18	30,5	
Baixo peso (2)	8	12,1	10	15,2	6	10,0	8	13,6	
Obesidade 1, 2 e 3 (3)	33	50,0	35	53,0	29	48,3	33	55,9	
Realiza alguma atividade física?									0,305
Não (2)	44	66,7	47	71,2	38	63,3	41	69,5	
Sim (1)	22	33,3	19	28,8	22	36,7	18	30,5	
Ganha peso entre as sessões superior a 4%									0,183
Não (1)	32	48,5	26	39,4	27	45,0	31	52,5	
Sim (2)	34	51,5	40	60,6	33	55,0	28	47,5	
Idade (anos)									
Média ± desvio padrão	63.6 ± 15.3								
Tempo que faz diálise (anos)									
Média ± desvio padrão (amplitude)	4.5 ± 5.3								
Mediana (1°–3° quartil)	2.3 (0.8–7.0)								
Tipo de cateter									
Central duplo lúmen	31		47,0	32	48,5	30	50,0	28	47,5
Fístula arteriovenosa	35	53,0	34	51,5	30	50,0	31	52,5	

Notas – ^a^: Percentuais obtidos com base no total de cada semana.

^b^: Teste de Friedmann test.

Foram também analisadas as informações sobre quedas e a classificação de risco pela escala. Na S1, 40,9% dos pacientes relataram de uma a três quedas no último ano, e 30,3% relataram quatro ou mais episódios, sem diferença estatística significativa entre as semanas. A maior parte das quedas ocorreu na residência ou em via pública, variando de 51,5% a 55,9%. Quanto à classificação de risco pela ERQUE_HD, o risco moderado predominou em todas as semanas, com proporções entre 80,3% e 83,3%. Sobre as quedas na última semana, não houve variação estatisticamente significativa ao longo do seguimento (p = 0,366); a maioria dos pacientes não sofreu quedas nesse período, variando de 71,7% na S3 a 79,7% na S4. Entre os que relataram quedas recentes, episódios entre uma e três foram os mais comuns, atingindo até 19,7% dos casos. Quanto à classificação de risco para quedas na última semana, a maioria dos respondentes foi classificada como de risco moderado.

Buscou-se identificar em que medida as características gerais do perfil da amostra estiveram associadas à ocorrência de quedas. Considerando a classificação original desse desfecho, tanto no último ano quanto na última semana, foram identificados resultados estatisticamente significativos.

Quanto às análises das ocorrências de quedas, foram observadas associações significativas no último ano com diversas variáveis clínicas. Na S3, a deficiência visual e auditiva foi associada a um maior número de episódios (≥4). A fadiga pós-diálise teve associação significativa na S1: pacientes sem eventos relataram menor fadiga, enquanto aqueles com mais episódios referiram maior limitação nas atividades após a diálise. A hipotensão intradialítica foi fortemente associada às quedas nas semanas S2 e S4, especialmente entre os que sofreram quatro ou mais episódios. Já a hipotensão pós-diálise foi significativa na S2 e ocorreu com maior frequência entre os pacientes que sofreram quedas. Na S3, a marcha comprometida foi associada a um maior número de episódios, enquanto a marcha preservada foi observada entre os que tiveram menos eventos. Por fim, o ganho de peso interdialítico (>4%) associou-se à ocorrência de quedas nas semanas S3 e S4, sendo mais comum entre pacientes que relataram tais episódios, enquanto aqueles sem quedas mantiveram peso estável entre as sessões.

Foram analisadas as distribuições absolutas e relativas das características que apresentaram diferenças estatisticamente significativas na comparação com a ocorrência de quedas na última semana. Os resultados revelaram associações estatisticamente relevantes com diversos fatores clínicos. A hipotensão intradialítica mostrou-se fortemente associada à ocorrência de quedas nas semanas S3 e S4: pacientes que não apresentaram tais eventos estavam associados à ausência de hipotensão—67,4% (n = 29) e 72,3% (n = 34), respectivamente—enquanto aqueles com episódios de queda, especialmente os com quatro ou mais, apresentaram forte associação com essa condição. Nas semanas S3 e S4, pacientes com quatro ou mais episódios apresentaram associação de 100,0% (n = 6) com a presença de hipotensão. A hipotensão pós-diálise também esteve relacionada à ocorrência de quedas na última semana, na S4, sendo mais comum entre os pacientes que relataram esse evento. O uso de dispositivos de auxílio à marcha foi significativamente associado à ocorrência de quedas, principalmente nas semanas S2 e S3, com destaque para os que relataram uso constante desses dispositivos. Além disso, a marcha comprometida também se associou a quedas na S3, enquanto a marcha normal foi predominante entre os pacientes que não apresentaram episódios.

Para avaliar a relação entre a classificação de risco da escala ERQUE_HD e a ocorrência de quedas, foi realizada uma comparação entre essas variáveis em cada semana, considerando tanto as quedas no último ano quanto na última semana. Inicialmente, a ocorrência de queda foi analisada em três categorias e, posteriormente, agrupada em uma variável dicotômica (queda: sim/não). Os resultados indicaram associação estatisticamente significativa entre a classificação de risco e a ocorrência de quedas no último ano. Pacientes com risco moderado foram majoritariamente associados à ausência de quedas, enquanto aqueles com risco elevado apresentaram associação significativa com quatro ou mais episódios. Essa associação permaneceu significativa mesmo após a dicotomização da variável, reforçando que a classificação de risco alto está relacionada à ocorrência de quedas no último ano, conforme apresentado na Tabela 4.

**Tabela 4:** Distribuição absoluta e relativa da ocorrência de quedas no último ano segundo a classificação de risco

Quedas no último ano	Classificação de risco ERQUE_HD^a^ (pontuações da escala para o último ano)				p^b^
	Risco moderado		Risco alto		
	n	%	n	%	
Semana 1					0,005
Nenhuma queda	19	35,8			
De 1 a 3 episódios	22	41,5	5	38,5	
4 ou mais episódios	12	22,6	8	61,5	
Semana 2					0,002
Nenhuma queda	19	34,5			
De 1 a 3 episódios	25	45,5	3	27,3	
4 ou mais episódios	11	20,0	8	72,7	
Semana 3					0,005
Nenhuma queda	15	30,0			
De 1 a 3 episódios	21	42,0	2	20,0	
4 ou mais episódios	14	28,0	8	80,0	
Semana 4					0,30
Nenhuma queda	14	28,6			
De 1 a 3 episódios	22	44,9	3	30,0	
4 ou mais episódios	13	26,5	7	70,0	
Queda no último ano (SIM/NÃO)					
Semana 1					0,013
Não	19	35,8			
Sim	34	64,2	13	100,0	
Semana 2					0,026
Não	19	34,5			
Sim	36	65,5	11	100,0	
Semana 3					0,054
Não	15	30,0			
Sim	35	70,0	10	100,0	
Semana 4					0,098
Não	14	28,6			
Sim	35	71,4	10	100,0	

Notas – ^a^Percentuais obtidos com base no total de cada classificação da ocorrência de queda no último ano.

^b^Teste exato de Fisher (simulação de Monte Carlo).

Outra análise comparou a ocorrência de queda na última semana com a classificação de risco. Foram observadas associações significativas: pacientes com classificação de risco alto apresentaram associação significativa com quatro ou mais episódios de queda no último ano. Tais resultados foram evidenciados nas semanas S2 e S3. Quando a comparação foi realizada entre a classificação de risco e a ocorrência de queda, considerando a resposta dicotômica, a diferença significativa se mostrou presente nas três semanas avaliadas.

Para avaliar a capacidade preditiva da escala em relação à ocorrência de queda no último ano, foi utilizada a modelagem de EEG, com regressão logística ajustada para o seguimento de quatro semanas. O desfecho considerado foi a ocorrência de queda (sim/não) e o modelo investigou também o impacto de covariáveis associadas à queda ou à classificação de risco. Entre os preditores com potencial significativo, destacaram-se: deficiência visual/auditiva, fadiga pós-diálise (p < 0,001), hipotensão intradialítica, hipotensão pós-diálise, marcha alterada e ganho de peso superior a 4%. Inicialmente, foram testados modelos individuais para cada preditor, a fim de analisar sua contribuição isolada na explicação da ocorrência de quedas no último ano.

A análise bivariada para os modelos de regressão logística univariada, utilizando o método EEG, para predizer/explicar a ocorrência de queda no último ano (OR e IC95%), demonstrou que a classificação de risco da escala ERQUE_HD tem capacidade preditiva significativa para quedas no último ano (p < 0,0001). Pacientes classificados como de alto risco apresentaram uma probabilidade 6,764 vezes maior de queda em comparação aos de risco moderado. Entre as covariáveis com potencial preditivo, destacaram-se a deficiência visual/auditiva (p = 0,006), com aumento de 1,856 vezes no risco de queda, e a fadiga pós-diálise (p = 0,003), que elevou o risco em 5,588 vezes entre pacientes que responderam positivamente às duas questões sobre fadiga. A hipotensão pós-diálise também foi significativa, com pacientes que aguardaram ao menos 30 minutos para ir embora apresentando 2,696 vezes mais chances de queda. Embora a hipotensão sem espera tenha sugerido risco, o resultado não foi estatisticamente significativo, provavelmente devido ao número reduzido de casos. O ganho de peso superior a 4% entre sessões foi outro fator relevante (p < 0,001), aumentando em 2,292 vezes o risco de quedas. Por fim, as variáveis hipotensão intradialítica e marcha não se mantiveram significativas, possivelmente devido à perda de efeito ao longo do seguimento.

Com base nos resultados alcançados nos modelos univariados (modelos individuais), as variáveis que apontaram resultado significativo para predizer/explicar a ocorrência de queda foram selecionadas para compor o modelo multivariado. Dessa forma, o modelo multivariado inicial teve como variáveis independentes a classificação de risco ERQUE_HD, deficiência visual/auditiva, doença óssea, fadiga, hipotensão pós-diálise e ganho de peso. De acordo com os resultados obtidos, o modelo ótimo, elencado como preditor da ocorrência de queda no último ano, foi formado pelos seguintes preditores: classificação de risco (risco elevado—OR; IC95%), fadiga pós-diálise (sim para as duas questões—OR; IC95%) e ganho de peso superior a 4% entre sessões (sim—OR; IC95%).

Destaca-se que a classificação de risco, como preditor da ocorrência de queda, teve seu poder de predição aumentado frente à presença da fadiga e do ganho de peso entre as sessões superior a 4%; no modelo simples, o OR para a classificação de risco elevado para ocorrência de queda foi estimada em 6,764, enquanto, no modelo múltiplo, o OR passou para 8,731, indicando um desempenho preditivo aumentado da variável de classificação de risco, conforme demonstrado Tabela 5.

**Tabela 5:** Modelo de regressão logística multivariada pelo método de estimação de equações generalizadas (EEG) para predizer/explicar a ocorrência de quedas no último ano

Características da amostra	OR para a ocorrência de quedas no último ano				
	Odds ratio^a^				Odds ratio^a^
	OR	IC95%^b^			
MODELO 2c					
Classificação de risco anual					
Risco moderado	1,0				
Risco alto	8,731	2,681	19,687		<0,00001
Fadiga pós-diálise: “Sentiu-se cansado após as sessões de hemodiálise?” “A fadiga limitou ou atrapalhou suas atividades diárias nos dias subsequentes?”					
Não para as 2 questões (0)	1,0				
Sim para uma questão (1)	3,113	1,135	8,545		0,053
Sim para as duas questões (2)	5,715	1,841	17,743		0,003
Ganho de peso entre as sessões superior a 4%					
Não (1)	1,0				
Sim (2)	2,415	1,161	5,026		<0,001

Notas – ^a^:Estimativas obtidas por equações de estimação generalizadas (EEG).

^b^:OR, odds ratio; IC95%, intervalo de confiança de 95% para o OR.

^c^:Desempenho do modelo logístico – matriz de confusão (porcentagem correta: 78,9%; acertos na ocorrência de quedas: 95,7% [176/184]; acertos na não ocorrência de quedas: 32,8% [45/67]). R^2^ Cox & Snell [0,288]. R^2^ Nagelkerke [0,419].F

Em relação ao desempenho do modelo, buscou-se investigar a robustez da acurácia dos resultados de classificação alcançados pela regressão logística sobre a classificação real observada na amostra para quedas no último ano. Empregou-se a técnica da área sob a curva ROC, na qual, além da estimativa da AUC para identificar a significância estatística da capacidade de acerto do modelo múltiplo, também foram reportados os valores de sensibilidade e especificidade.

De acordo com o resultado alcançado, verificou-se que a AUC foi estimada em 0,817 (IC95%: 0,745–0,889; p < 0,001). Na análise da sensibilidade e da especificidade do modelo preditivo para ocorrência de quedas no último ano, as estimativas foram de 0,876 e 0,684, respectivamente. Destaca-se que as estimativas obtidas pela curva ROC ocorreram com base nas pontuações da escala ERQUE_HD, em relação à classificação do modelo preditivo, sendo o ponto de corte para ocorrência de quedas definido como pontuações acima de 23 (*cutoff point* >23).

A análise envolvendo a acurácia alcançada no modelo univariado, que utilizou exclusivamente a classificação de risco para predizer a ocorrência de queda, teve como estimativa para a curva ROC o valor de 0,702 (IC95%: 0,614–0,811), indicando que a acurácia também se mostrou relevante para a predição da ocorrência de quedas no último ano. Quanto às estimativas de sensibilidade e especificidade do modelo preditivo univariado, os valores foram de 0,814 e 0,568, respectivamente.

## Discussão

O desenvolvimento e validação da ERQUE_HD trata-se de uma tarefa complexa, exigindo conhecimentos científicos aliados a desfechos e a uma gama de conhecimentos provenientes de especialistas e do público que realiza essa terapia. Os resultados deste estudo evidenciaram diversos fatores de risco para quedas no público-alvo. Após as diversas etapas realizadas, os itens foram definidos, culminando na versão final da escala, com um IVC de 0,95, reforçando que o conteúdo da ferramenta foi validado de acordo com os valores recomendados^9,13^.

A relevância deste instrumento torna-se ainda mais evidente no cenário global de envelhecimento populacional. Diante dessa situação, a inclusão da idade como fator de risco na escala foi aplicada a partir das evidências identificadas nos grupos focais e na revisão integrativa. Reforçando esse cenário, a Organização Mundial da Saúde (OMS) relata que, até 2050, mais de 2 bilhões de pessoas terão mais de 60 anos^17^. Estima-se que, em um período de doze meses, um terço da população idosa com 65 anos ou mais sofrerá pelo menos uma queda, sendo que metade desses indivíduos apresentará episódios recorrentes^18^.

Nesse contexto, de acordo com o Censo Brasileiro de Diálise 2023, destaca-se que 62,5% dos pacientes em hemodiálise tinham entre 20 e 64 anos, e os 33,6% restantes tinham mais de 65 anos^3^. Ao analisar os resultados desta pesquisa, observa-se que 42,03% dos participantes em hemodiálise possuem mais de 65 anos, um valor superior ao reportado pelo censo. Essa discrepância pode ser atribuída, em parte, ao número reduzido de participantes da amostra atual, comparado ao número estudado pelo censo.

Esta pesquisa revelou que 76% dos participantes em hemodiálise apresentaram quedas no último ano, um percentual bem acima das estimativas da literatura, como, por exemplo, um estudo que reportou uma incidência de 23,3%^19^. Essa diferença pode ser atribuída, em grande parte, à metodologia empregada neste estudo, especialmente no que se refere à busca ativa por informações sobre quedas, realizada por meio de entrevistas diretas e detalhadas com os pacientes.

Em contrapartida, muitos estudos presentes na literatura se baseiam em dados secundários ou em notificações espontâneas, o que pode resultar em subnotificação de eventos como quedas, sobretudo quando estas não resultam em lesões graves ou internações. Assim, os resultados deste estudo reforçam a importância de estratégias ativas de monitoramento e investigação sistemática das quedas entre pacientes em hemodiálise, uma vez que a alta prevalência observada indica um problema de saúde significativo e potencialmente negligenciado. Um estudo relatou que a busca ativa resultou em uma prevalência oito vezes maior quando comparada à notificação espontânea, reiterando sua importância como método de monitoramento epidemiológico para um diagnóstico situacional realista, sendo um instrumento fundamental para a segurança do paciente^20^.

Ainda que estudos apontem uma forte associação entre o uso de determinados medicamentos e o aumento do risco de hipotensão e quedas, especialmente os anti-hipertensivos e aqueles que atuam no sistema nervoso central, como antiparkinsonianos, psicotrópicos, psicoanalépticos e antiepilépticos^21^, não há evidências conclusivas de correlação significativa na prática clínica. No entanto, é fundamental que, sempre que possível, se obtenha um levantamento completo dos medicamentos utilizados pelos pacientes, a fim de possibilitar um manejo mais eficaz. Na ERQUE_HD, não existe pontuação específica para medicamentos relacionados ao risco de quedas; 93,9% dos participantes utilizam mais de dois medicamentos por dia, reforçando a importância de incluir um item que avalie, de maneira geral, o uso ou não de medicamentos, sem atribuição de pontuação específica por fármaco utilizado.

No que diz respeito ao histórico de saúde, estudos anteriores indicam uma forte relação entre diabetes e risco de quedas. Uma das complicações mais comuns do diabetes é a neuropatia, tanto sensitiva quanto motora, que pode ser caracterizada por sintomas como perda da propriocepção, atrofia e fraqueza dos músculos intrínsecos do pé, levando a deformidades do pé e anormalidades no padrão da marcha, o que aumenta significativamente o risco de quedas^22^. No entanto, após a aplicação da escala ERQUE_HD, observou-se que tanto a presença de diabetes quanto o possível déficit motor—um fator relacionado à progressão da doença—não apresentaram valor de significância estatística quando comparados a outras complicações associadas à diálise. Apesar disso, considerando os estudos analisados e as discussões dos grupos focais, optou-se por pontuar, no escore, os pacientes com diagnóstico confirmado dessa condição.

Um estudo avaliou pacientes com doença mineral óssea decorrente da doença renal crônica, os quais apresentaram alto risco de quedas e maior comprometimento dos aspectos físicos e da qualidade de vida^23^. Da mesma forma, a deficiência visual/auditiva e a doença óssea, quando avaliadas isoladamente, apresentaram alta significância; entretanto, quando concomitantes a outros fatores de risco, sua relevância foi significativamente menor. Esses fatores foram incluídos na avaliação da escala.

Outras complicações podem ocorrer durante a terapia e estar associadas a outros fatores de risco para quedas, como cãibras, dores lombares, febre, calafrios, hipoglicemia e hipotensão sintomática intradialítica^24^. A ocorrência de hipotensão sintomática depende, provavelmente, da quantidade e da taxa de remoção de fluido intravascular durante o procedimento. Em resposta à ultrafiltração (UF), uma redução significativa no volume circulante leva à diminuição do enchimento atrial, à redução do débito cardíaco e, por fim, à hipotensão, a menos que haja fluido intersticial suficiente para o reabastecimento intravascular^25^. Apesar de serem fatores extremamente relevantes para o monitoramento durante o tratamento de hemodiálise, a fim de possibilitar intervenções adequadas e proporcionar melhor qualidade de vida aos pacientes, eles não se mostraram, de fato, preditores da ocorrência de quedas.

A hipotensão pós-diálise está associada às quedas, sugerindo que a instabilidade hemodinâmica no período pós-sessão é um fator crítico a ser monitorado^23^. Ainda, o ganho de peso interdialítico elevado, indicativo de sobrecarga hídrica, também apresentou relação com o risco aumentado de eventos, possivelmente por contribuir para alterações no equilíbrio e na pressão arterial. Um estudo revelou que 32% dos eventos de quedas em pacientes submetidos à hemodiálise estão relacionados à variação de peso durante o tratamento, identificando essa mudança como um fator de risco significativo. A retirada excessiva de líquidos está frequentemente associada a sintomas como tontura e fraqueza, que podem preceder os episódios de queda^19^. Esses resultados corroboram os achados deste estudo, no qual 45,3% dos participantes relataram hipotensão pós-diálise e 51,5% apresentaram ganho de peso superior a 4% entre as sessões. Consequentemente, 72,3% dos pacientes relataram quedas na residência ou na rua após a sessão de hemodiálise. Esses eventos já foram associados às quedas e, no presente estudo, evidenciam diretamente a variação de peso sofrida pelo paciente durante a sessão de HD, a fadiga e a hipotensão intradialítica como fatores diretamente relacionados ao risco de quedas.

O desenvolvimento de uma escala de avaliação de risco para quedas em hemodiálise constitui uma estratégia relevante para o fortalecimento das ações de segurança do paciente pertencente a esse grupo de risco. A adoção desse instrumento visa proporcionar uma análise mais abrangente e precisa dos fatores de risco individuais, considerando aspectos clínicos, funcionais e ambientais que predispõem o paciente a esse desfecho. A aplicação sistemática da escala permitiu a identificação precoce dos pacientes mais vulneráveis. Adicionalmente, espera-se que a escala contribua significativamente para a elaboração de intervenções preventivas direcionadas e personalizadas, bem como para a padronização dos cuidados prestados pela equipe multiprofissional, a redução da incidência de quedas, a minimização de complicações associadas e, consequentemente, a melhoria da qualidade de vida dos pacientes em terapia dialítica.

Este estudo apresenta como limitação a aplicação da escala ERQUE_HD apenas em um centro de diálise, sendo desejável sua aplicação em outros centros com perfis de pacientes diferentes.

## Conclusões

A escala ERQUE_HD é preditiva do risco de quedas, principalmente nos pacientes classificados como de alto risco para esse evento. Espera-se que a escala auxilie na aplicação de medidas preventivas nesse público específico, contribuindo para a redução da ocorrência desse evento adverso e para a promoção de um cuidado mais seguro aos pacientes em hemodiálise.
